# Loss of TMEM106B exacerbates C9ALS/FTD DPR pathology by disrupting autophagosome maturation

**DOI:** 10.3389/fncel.2022.1061559

**Published:** 2022-12-16

**Authors:** Claudia S. Bauer, Christopher P. Webster, Allan C. Shaw, Jannigje R. Kok, Lydia M. Castelli, Ya-Hui Lin, Emma F. Smith, Francisco Illanes-Álvarez, Adrian Higginbottom, Pamela J. Shaw, Mimoun Azzouz, Laura Ferraiuolo, Guillaume M. Hautbergue, Andrew J. Grierson, Kurt J. De Vos

**Affiliations:** ^1^Sheffield Institute for Translational Neuroscience (SITraN), Department of Neuroscience, University of Sheffield, Sheffield, United Kingdom; ^2^Neuroscience Institute, University of Sheffield, Sheffield, United Kingdom

**Keywords:** C9orf72, DPR, autophagy, ALS/FTD, TMEM106B

## Abstract

Disruption to protein homeostasis caused by lysosomal dysfunction and associated impairment of autophagy is a prominent pathology in amyotrophic lateral sclerosis and frontotemporal dementia (ALS/FTD). The most common genetic cause of ALS/FTD is a G4C2 hexanucleotide repeat expansion in *C9orf72* (C9ALS/FTD). Repeat-associated non-AUG (RAN) translation of G4C2 repeat transcripts gives rise to dipeptide repeat (DPR) proteins that have been shown to be toxic and may contribute to disease etiology. Genetic variants in *TMEM106B* have been associated with frontotemporal lobar degeneration with TDP-43 pathology and disease progression in C9ALS/FTD. *TMEM106B* encodes a lysosomal transmembrane protein of unknown function that is involved in various aspects of lysosomal biology. How *TMEM106B* variants affect C9ALS/FTD is not well understood but has been linked to changes in TMEM106B protein levels. Here, we investigated TMEM106B function in the context of C9ALS/FTD DPR pathology. We report that knockdown of TMEM106B expression exacerbates the accumulation of C9ALS/FTD-associated cytotoxic DPR proteins in cell models expressing RAN-translated or AUG-driven DPRs as well as in C9ALS/FTD-derived iAstrocytes with an endogenous G4C2 expansion by impairing autophagy. Loss of TMEM106B caused a block late in autophagy by disrupting autophagosome to autolysosome maturation which coincided with impaired lysosomal acidification, reduced cathepsin activity, and juxtanuclear clustering of lysosomes. Lysosomal clustering required Rab7A and coincided with reduced Arl8b-mediated anterograde transport of lysosomes to the cell periphery. Increasing Arl8b activity in TMEM106B-deficient cells not only restored the distribution of lysosomes, but also fully rescued autophagy and DPR protein accumulation. Thus, we identified a novel function of TMEM106B in autophagosome maturation via Arl8b. Our findings indicate that *TMEM106B* variants may modify C9ALS/FTD by regulating autophagic clearance of DPR proteins. Caution should therefore be taken when considering modifying TMEM106B expression levels as a therapeutic approach in ALS/FTD.

## Introduction

A hexanucleotide GGGGCC (G4C2) repeat expansion in the *C9orf72* gene is the most common cause of amyotrophic lateral sclerosis (ALS) and frontotemporal dementia (FTD) (DeJesus-Hernandez et al., 2011; Renton et al., 2011). Three non-exclusive mechanisms have been proposed by which the expansion may cause disease, namely RNA toxicity induced via sequestration of nuclear proteins, haploinsufficiency, and protein toxicity deriving from repeat associated non-AUG (RAN) translation of sense and antisense repeat transcripts into five types of dipeptide repeat (DPR) proteins, poly(GA), poly(GR), poly(GP), poly(PR), and poly(PA) [Reviewed in Balendra and Isaacs (2018)].

In healthy tissues, protein aggregates are cleared by autophagy, a lysosomal degradation pathway responsible for the bulk clearance of cytoplasmic components. Autophagy receptors such as SQSTM1/p62 recognize protein aggregates and direct them to nascent autophagosomes which upon completion fuse with lysosomes to allow substrate degradation (Lamark and Johansen, 2012). Analysis of post-mortem CNS tissues from C9ALS/FTD patients shows that DPR protein inclusions are found throughout the brain and recruit the autophagy receptor SQSTM1/p62 (Cooper-Knock et al., 2012; Mann et al., 2013), indicating failure to clear them by autophagy. We and others have shown that the C9orf72 protein is a regulator of autophagy (Sellier et al., 2016; Webster et al., 2016). Consistent with C9orf72 haploinsufficiency we found that basal autophagy is reduced in C9ALS/FTD patient-derived iNeurons (Webster et al., 2016). Moreover, it has been shown that impaired autophagy due to loss of C9orf72 increases levels of DPR proteins and thereby exacerbates DPR protein aggregate-based toxicity *in vitro* and in mice expressing human transgenes carrying the repeat expansion (Boivin et al., 2020; Zhu et al., 2020). We have shown that C9orf72 plays a cell-autonomous role in the regulation of neurotransmission at excitatory synapses and that C9orf72 haploinsufficiency leads to loss of synapses and synaptic dysfunction (Bauer et al., 2022). Synaptic loss has been also observed in a gain-of-function poly(GR) (80-repeat) mouse model (Choi et al., 2019), and overexpression of GA DPRs has been shown to cause damage to synaptic vesicle release in cortical neurons (Jensen et al., 2020). Thus, a model emerges in which C9orf72 haploinsufficiency-mediated impairments synergize with DPR protein toxicity in a multi-hit mechanism.

Genetic variants in *transmembrane protein 106 B* (*TMEM106B*) are genetic modifiers of frontotemporal lobar degeneration with TDP-43 pathology (FTLD-TDP) in patients with pathological G4C2 hexanucleotide expansions in *C9orf72* (Van Deerlin et al., 2010; Vass et al., 2011; Gallagher et al., 2014; van Blitterswijk et al., 2014). Furthermore, *TMEM106B* might modify disease progression in C9ALS/FTD patients presenting with ALS (Deming and Cruchaga, 2014; van Blitterswijk et al., 2014).

The TMEM106B protein is a glycosylated, single pass, type 2 transmembrane protein that localizes to the endo-lysosomal compartment (Lang et al., 2012). TMEM106B has been implicated in various aspects of lysosomal biology, including lysosome size, acidification, trafficking, and stress signaling but the precise molecular function of TMEM106B is still unclear (Chen-Plotkin et al., 2012; Brady et al., 2013; Schwenk et al., 2014; Stagi et al., 2014; Jun et al., 2015; Busch et al., 2016; Kundu et al., 2018; Lüningschrör et al., 2020; Zhou et al., 2020). How *TMEM106B* variants modify C9ALS/FTD has not been resolved, but evidence suggests that the single nucleotide polymorphisms (SNPs) associated with FTLD-TDP modulate TMEM106B protein levels; the only coding variant rs3173615 C/G (p.T185S), which is in linkage disequilibrium with the top risk variant rs1990622 T/C, appears to influence TMEM106B turnover rates: S185 in TMEM106B disrupts an N-glycosylation site at N183 and this causes accelerated degradation and consequently lower expression levels of TMEM106B/S185 compared to TMEM106B/T185 (Van Deerlin et al., 2010; Busch et al., 2013; Nicholson et al., 2013; Satoh et al., 2014).

Here we investigated TMEM106B function in the context of C9ALS/FTD. We used cell models expressing RAN-translated or AUG-driven DPRs as well as C9ALS/FTD patient-derived iAstrocytes with an endogenous G4C2 expansion to evaluate if loss of TMEM106B function affects DPR proteins. We further investigated TMEM106B function in the autophagy pathway and its relation to DPR protein clearance.

We demonstrate that reduced levels of TMEM106B cause accumulation of C9ALS/FTD-associated DPR proteins in cell models and patient-derived iAstrocytes by impairing autophagy. We further identify a novel role of TMEM106B in autophagosome to autolysosome maturation via regulation of Arl8b-mediated anterograde trafficking of lysosomes. Finally, we show that restoration of autophagosome maturation rescues DPR protein accumulation. Thus, our data shed new light on some aspects of the disease modifying effects of TMEM106B in C9ALS/FTD.

## Materials and methods

### Plasmids

pCI-Neo (Promega) was used as an empty vector control. pCI-Neo-mCherry-N was created by inserting mCherry-(GGGGS)2, generated by PCR using Phusion High Fidelity enzyme (NEB) from pmCherry-N1 (Takara Bio), into the Nhe1/Xho1 sites of pCI-Neo. Human TMEM106B cDNA (clone IRATp970G1031D) was obtained from Source BioScience LifeSciences. TMEM106B/T185 cDNA was amplified by PCR using 5′-CTCGAGctttctgctgacttcaactcctc-3′ and 5′-GCGGCCGCttctttaaatccatctcttccagttt-3′ primers and subcloned into the Xho1 and Not1 restriction sites of the pCI-Neo-myc vector.

Arl8b was tagged with a carboxyterminal mVenus tag by subcloning from pCMV6-Entry-Arl8b-MycDDK (Origene), into pCI-Neo-C-mVenus using BamH1/MluI restriction sites. pCI-Neo-C-mVenus was created by inserting (GGGGS)2-mVenus, generated by PCR from prSETB-mVenus (a gift from Dr. Atsushi Miyawaki, RIKEN, Japan), into pCI-Neo using the SacII/Not1 restriction sites.

pcDNA3.1-G4C2 × 45-3xV5 and pcDNA3.1-C4G2 × 43-3xV5 are described in Supplementary Figures 1, 2, respectively (Castelli et al., 2021).

AUG-driven synthetic, codon-optimized, AcGFP1-tagged 6 repeat, and untagged 36 and 100 repeat poly(PR), poly(GR) or poly(GA) DPR constructs in pcDNA3.1 were a gift from Prof. Adrian Isaacs (UCL, London, UK) and have been described previously (Mizielinska et al., 2014). To generate V5-tagged 36 and 100 repeat poly(PR), poly(GR) or poly(GA) DPRs the untagged constructs were subcloned using BamHI/NotI into pCI-Neo-V5-N opened with BclI/NotI.

mCherry-EGFP-LC3b was a gift from Prof. Terje Johansen, University of Tromsø, Norway (Pankiv et al., 2007). All constructs were confirmed by Sanger sequencing.

### Cell culture and transfection

HEK293 and HeLa cells were cultured in Dulbecco’s modified Eagle’s medium (DMEM, Sigma) supplemented with 10% FBS (Biosera) and 1 mM sodium pyruvate (Sigma) in a humified, 5% CO_2_ atmosphere at 37°C. Cells were transfected with plasmid DNA using Lipofectamine 2000 (Invitrogen) or TurboFect*™* reagent (Thermo Fisher Scientific) according to the manufacturer’s instructions or polyethylenimine (PEI) (stock 1 mM; 3 μl/μg plasmid). Cells were used for experiments 24-48 h after transfection.

HeLa cells were siRNA transfected using Lipofectamine RNAiMax (Invitrogen) according to the manufacturer’s instructions. Cells were used for experiments 4 days after siRNA transfection.

### siRNA and shRNA

Non-targeting control siRNA and targeting siRNA was purchased from Sigma-Aldrich/Merck Life Science or Integrated DNA Technologies (IDT). The siRNA sequences were as follows: TMEM106B#2: guacucaugaugcaaguuauu; Rab7A#1: cagacugcugcguucugguuu, Rab7A#2 cugaaccuau caaacuggauu. Rab7A siRNAs were pooled in a 1:1 ratio.

For lentiviral knockdown of TMEM106B, TMEM106B shRNA (gtactcatgatgcaagtta) and a control shRNA (ccta aggttaagtcgccctcg) were cloned into pLV-hPGK_EFGP:T 2A:Puro_U6_shRNA and packaged into lentiviral particles by Vectorbuilder.

### iNPC production and iAstrocyte differentiation

Skin biopsies were obtained from the forearm of subjects after informed consent, in accordance with guidelines set by the local ethics committee (Study number STH16573, Research Committee reference 12/YH/0330). Fibroblast cell cultures were established in cell culture medium (Lonza) supplemented with 10% FCS (Labtech), 2 mM glutamine, 50 μg/ml uridine, vitamins, amino acids and 1 mM sodium pyruvate in a humified, 5% CO_2_ atmosphere at 37°C. iAstrocytes were differentiated as previously described (Meyer et al., 2014). Briefly, induced neural progenitors (iNPCs) were cultured in Dulbecco’s modified Eagle medium (DMEM) containing 1% N2 supplement (Life Technologies), 1% B27 supplement and 20 ng/ml fibroblast growth factor-2 (Preprotech). INPCs were differentiated into induced astrocytes (iAstrocytes) on 10 cm dishes coated with fibronectin (5 μg/ml, Millipore) by culturing in DMEM with 10% FBS and 0.3% N2. INPCs were differentiated to iAstrocytes over 8 days.

### Lentiviral transduction of iAstrocytes

On day 3 of iAstrocyte differentiation control and C9ALS patient-derived iAstrocytes were seeded to 6 well plates coated with fibronectin (5 μg/ml, Millipore) at a density of 90,000 cells per well. 24 h after seeding, on day 4 of differentiation, C9ALS patient cells were transduced with EGFP-shCtrl or EGFP-shTMEM lentiviruses (VectorBuilder) at a multiplicity of infection (MOI) of 5 transducing units (TU)/cell in DMEM with 10% FBS and 0.3% N2, supplemented with 1 μg/ml polybrene. 24 h post transduction, on day 5 of differentiation, the transduction media was replaced with fresh DMEM with 10% FBS and 0.3% N2. On day 7 of differentiation, cells were given a further 50% media change. Cells were lysed directly in RIPA buffer 4 days post transduction, on day 8 of differentiation, and protein concentration measured by BCA assay ready for analysis of poly(GP) DPRs via MSD ELISA.

### Electrochemiluminescent ELISA for poly(GP)

Poly(GP) levels were determined by a Meso Scale technology sandwich ELISA using the MSD QUICKPLEX SQ120 platform (Meso Scale Technology). The purified rabbit polyclonal capture antibody was a kind gift from Prof. Adrian Isaacs (UCL, UK), the detection antibody (Proteintech: 24494-1-AP) was biotinylated in house and the calibrant peptide (GP)7 (Biogene) was serially diluted to generate a standard curve. Each sample was prepared in ice cold RIPA buffer (50 mM Tris–HCl pH 6.8, 150 mM NaCl, 1 mM EDTA, 1 mM EGTA, 2% (w/v) SDS, 0.5% (w/v) deoxycholic acid, 1% (w/v) Triton X-100, and protease inhibitor cocktail (Thermo Scientific) to 1.5 mg/ml then mixed 50:50 with EC buffer [NaH_2_PO_4_ 5mM, Na_2_HPO_4_ 20mM, NaCl 400mM, EDTA 2.5mM, CHAPS 0.05%, BSA 0.2%, Block Ace 0.4%, NaN_3_ 0.05%, PMSF 1mM, protease inhibitor cocktail (Calbiochem)], testing duplicate wells of 50 μl each.

Briefly, multi-array plates were coated overnight with 30 μl capture antibody (2 μg/ml) in Tris buffered saline (TBS) at 4°C. Plates were then washed in TBS + 0.2% Tween-20 (TBST) and blocked for 2 h in 3% non-fat milk in TBST at room temperature (RT) shaking at 700 rpm, before being washed in TBST and incubated with calibrant or samples at 4°C overnight at 700 rpm. The plates were washed, incubated for 2 h at RT 700rpm with 25 μl biotinylated detection antibody (2 μg/ml) and 0.5 ng/ml SULFO-TAG streptavidin R32AD-1 in blocking buffer. Following the final wash, 150 μl 2x read buffer R92TD was added and then read.

### SDS-PAGE and immunoblotting

Cells were harvested in trypsin/EDTA (Lonza) and pelleted at 400 × *g* for 4 min. Pellets were washed once with phosphate buffered saline (PBS). Cells were lysed on ice for 30 min in ice-cold RIPA buffer [50 mM Tris HCl pH 6.8, 150 mM NaCl, 1 mM EDTA, 1 mM EGTA, 0.1% (w/v) SDS, 0.5% (w/v) deoxycholic acid, 1% (w/v) Triton X-100 and protease inhibitor cocktail (ThermoFisher Scientific)]. Lysates were clarified at 10,000 x g for 30 min at 4°C. Protein concentration was measured by Bradford Protein assay (Bio-Rad). All DPR expressing cells were harvested directly into 2x concentrated Laemmli loading buffer.

Proteins were separated by SDS-PAGE and transferred to nitrocellulose membranes (Whatmann) by electroblotting (Bio-Rad). After transfer, membranes were blocked for 1 h at room temperature in Tris-buffered saline (TBS) with 5% fat-free milk powder (Marvel or Sainsbury’s) and 0.1% Tween-20. Membranes were incubated with primary antibodies in blocking buffer for 1 h at room temperature or overnight at 4°C. Membranes were washed 3 times for 10 min in TBS with 0.1% Tween-20 before incubation with secondary antibodies in block buffer for 1 h at room temperature.

Secondary antibodies used for immunoblotting were horseradish peroxidase (HRP)-coupled goat anti-rabbit and goat anti-mouse IgG (Dako, Agilent Technologies LDA, London, UK; 1:5,000), or near-infrared fluorescent IRDye 680RD or 800CW Donkey anti-mouse or rabbit IgG (H + L) (926-68072; LI-COR Biosciences, 1:5,000) or Alexa Fluor 680 donkey anti-mouse IgG and Alexa Fluor 790 donkey anti-rabbit IgG (Jackson ImmunoResearch, Stratech Scientific Ltd., Ely, UK; 1:50,000). In case HRP-coupled antibodies were used as secondary antibodies, after washing, membranes were prepared for chemiluminescent signal detection with SuperSignal West Pico Chemiluminescent substrate (ThermoFisher Scientific) according to the manufacturer’s instructions.

Chemiluminescent signals were detected on ECL film (GE Healthcare), or using a SynGene Gbox Imager or an Odyssey^®^ Fc imaging system (LI-COR Biosciences). Fluorescent signals were detected using an Odyssey^®^ Fc imaging system. Signal intensities were quantified using ImageJ/Fiji (Abramoff et al., 2004; Schindelin et al., 2012) or ImageStudio™ (LI-COR Biosciences).

### Antibodies

Primary antibodies used were as follows:

Mouse anti-α-Tubulin (DM1A, Sigma, WB: 1:10,000)
Rabbit anti-Arl8b (13049-1-AP, ProteinTech, WB: 1:1,000)
Rabbit anti-C9orf72 (25757-1-AP, ProteinTech, WB: 1:500)
Mouse anti-Flag (M2, Sigma, WB: 1:2,000)
Rabbit anti-GAPDH (14C10, Cell Signaling, WB: 1:2,000)
Mouse anti-GFP (JL8, Clontech, WB: 1:5,000)
Mouse anti-HA (HA-7, Sigma, WB: 1:2,000)
Rabbit anti-HA (H6908, Sigma, IF: 1:1,000)
Mouse anti-LAMP2A (H4B4, Santa Cruz, IF: 1:250-500)
Rabbit anti-LC3 (2220, Novus Biologicals, WB: 1:1,000)
Mouse anti-Myc (9B11, Cell Signaling, WB: 1:2,000, IF: 1:1,000)
Rabbit anti-Myc (9106, Abcam, IF: 1:1,000)
Mouse anti-p62 (610833, BD Biosciences, WB: 1:1,000, IF: 1:1,000)
Rabbit anti-p62 (18420-1-AP, ProteinTech, IF: 1:500)
Mouse anti-poly(GA) (kindly provided by Prof. Dieter Edbauer, DZNE, Muenchen Germany, WB: 1:500)
Rabbit anti-poly(GR) (23978-1-AP, ProteinTech, WB: 1:500)
Rabbit anti-poly(PR) (23979-1-AP, ProteinTech, WB: 1:500)
Rabbit anti-poly(GP) (24494-1-AP, ProteinTech, WB: 1:500)
Rabbit anti-Rab7 (ab137029, Abcam, WB: 1:500)
Rabbit anti-SMCR8 (ab202283, Abcam, WB: 1:1,000)
Mouse anti-TMEM106B (60333-1-Ig, Proteintech, WB: 1:1,000)
Mouse anti-V5 (R960, Invitrogen, WB: 1:5,000, IF: 1:1,000)

### Immunofluorescence

Immunostaining was performed as described previously (De Vos et al., 2005). Briefly, cells on glass coverslips were fixed with 3.7% formaldehyde in phosphate buffered saline (PBS) for 20 min at room temperature. After washing with PBS, residual formaldehyde was quenched by incubation with 50 mM NH_4_Cl in PBS for 10 min at room temperature, followed by a second round of washing with PBS. Subsequently, the cells were permeabilized by incubation with 0.2% Triton-X100 in PBS for 3 min. Triton-X100 was removed by washing with PBS. After permeabilization, the cells were incubated with PBS containing 0.2% fish gelatine (PBS/F) for 30 min at room temperature and then with the primary antibody in PBS/F for 1 h at RT. After washing with PBS/F, the cells were incubated with secondary antibody in PBS/F for 45 min at room temperature and stained with Hoechst 33342. After a final wash, the samples were mounted in fluorescence mounting medium (Dako).

Secondary antibodies used for immunofluorescence were Alexa fluorophore (488, 568, or 633)-coupled goat or donkey anti-mouse IgG, Alexa fluorophore (488, 568, or 633)-coupled goat or donkey anti-rabbit IgG, Alexa fluorophore (488, 568, 647)-coupled donkey anti-goat IgG (All from Invitrogen, ThermoFisher Scientific; 1:500).

### Microscopy

Images were recorded using appropriate filters (Omega Optical and Chroma Technology) using MicroManager 1.4 software (Edelstein et al., 2014) on a Zeiss Axioplan 2 microscope fitted with a Hamamatsu C4880-80 or Retiga R3 (QImaging) CCD camera, PE-300 LED illumination (CoolLED) and a 63x, 1.4NA Plan Apochromate objective (Zeiss) and using MetaMorph software (Molecular Devices) on an Olympus IX73 equipped with a Zyla4.2 sCMOS camera (Andor), SpectraX light engine (Lumencor) and OptoLED (Cairn Research) illumination, and 60x, 1.35NA Universal Plan Super Apochromat and 40x, 1.35NA Universal Apochromat objectives (Olympus).

### Lysosomal function assays

Cells on coverslips were loaded for 60 min in a 5% CO_2_ atmosphere at 37°C with 1 μM LysoSensor Green DND-189 (Invitrogen) in culture medium before being mounted onto a temperature-controlled microscope stage (QE-2 Quick Exchange Platform; Warner Instruments). Imaging was performed at 37°C in physiological buffer (145 mM NaCl_2_, 5 mM NaHCO_3_, 2 mM KCl, 2.5 mM MgCl_2_, 1 mM CaCl_2_, 10 mM HEPES, 20 mM glucose, pH 7.4/NaOH) using MetaMorph software (Molecular Devices). Cathepsin activity was imaged in live cells in physiological buffer at 37°C using the Magic Red Cathepsin B kit (Bio-Rad Laboratories) according to the manufacturer’s protocol.

### Image analysis

Image analysis was performed using ImageJ/Fiji (Abramoff et al., 2004; Schindelin et al., 2012) or CellProfiler™ 3.1.9 (McQuin et al., 2018). Where possible operators where blinded to the identity of the samples imaged and analyzed.

Lysosomes were classified as clustered or dispersed based on the distribution of the LAMP2A signal (measured in the 488 nm channel) relative to the nucleus (Hoechst 33342). Where possible, the cells for analysis were selected based on fluorescence in the 568 nm channel indicating co-transfection.

Lysosensor, Magic Red and V5-tagged DPR protein staining was quantified by measuring the mean fluorescence intensity per cell using ImageJ/Fiji.

Analysis of SQSTM1/p62 levels was automated using CellProfiler™ 3.1.9. Hoechst-labeled nuclei identified using the Minimum Cross Entropy thresholding method were used as seeds to identify cells by propagation. The cytoplasm was defined by masking the cell objects using the nuclei objects. The mean intensity of the SQSTM1/p62 channel was measured in the cytoplasm using the MeasureObjectIntensity module. In case of rescue experiments, transfected cells were identified based on the TMEM106B or Arl8b fluorescence intensity (minimum IntegratedDensity value of 1,000).

mCherry-EGFP-LC3 puncta were counted in single cells using the Particle Analysis facility of ImageJ/Fiji. Where possible, the cells for analysis were selected based on fluorescence in the other channel, or else the samples were blinded to the operator. Images were filtered using a Hat filter (7 × 7 kernel) (De Vos and Sheetz, 2007) to extract puncta and thresholded such that the visible puncta within the cell were highlighted, but no background was included. The result of thresholding was further checked against the original image to ensure no background signal was identified as puncta or signals were lost. In case of mCherry-EGFP-LC3, puncta in the red and green channels were counted separately. Red-only puncta were determined by subtracting the green puncta from the red puncta.

### Statistical analysis

Calculations and statistical analysis were performed using Excel (Microsoft Corporation, Redmond, WA), and Prism 8 or 9 software (GraphPad Software Inc., San Diego, CA). Details of statistical analysis can be found in the figure legends.

## Results

### Loss of TMEM106B leads to accumulation of C9ALS/FTD DPR proteins

**FIGURE 1:**
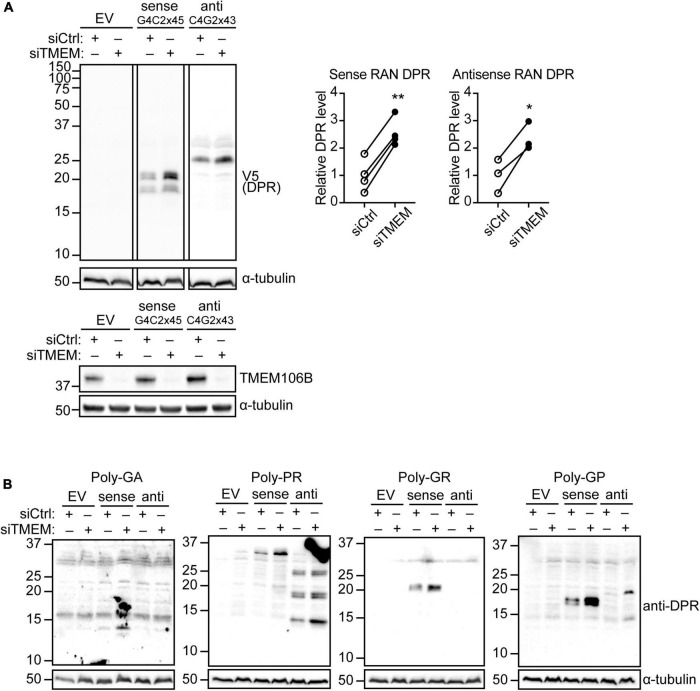
Knockdown of TMEM106B leads to accumulation of RAN translated C9ALS/FTD DPR proteins. HeLa cells were treated with non-targeting control (siCtrl) or TMEM106B-targeted siRNA (siTMEM) for 4 days before being transfected with empty vector (EV) or with 45 uninterrupted sense GGGGCC repeats (G4C2 × 45, Sense) or 43 uninterrupted antisense CCCCGG repeats (C4G2 × 43, Anti) with V5 tags in all three frames downstream of the repeats. **(A)** The levels of RAN translated DPR proteins were determined by immunoblot using anti-V5 antibodies. α-Tubulin indicates equal loading of samples. DPR levels were normalized to α-Tubulin and presented relative to siCtrl (unpaired *t*-test: *P ≤ 0.05, **P ≤ 0.01; Sense RAN DPR *N* = 4, Antisense RAN DPR *N* = 3 experiments). Knockdown of TMEM106B was confirmed by immunoblot using a TMEM106B antibody. **(B)** RAN translation was confirmed by immunoblot using DPR-specific poly(GA), poly(GR), poly(GP), and poly(PR) antibodies.

Reminiscent of ALS/FTD pathology, TMEM106B deficiency causes accumulation of ubiquitinated proteins and SQSTM1/p62 as well as phosphorylated TDP-43 in the brain and spinal cord of mouse models (Feng et al., 2020, 2022; Lüningschrör et al., 2020) and is associated with increased TDP-43 proteinopathy in human ALS brain (Mao et al., 2021). DPR proteins have emerged as an important driver of toxicity in C9ALS/FTD [Reviewed in Balendra and Isaacs (2018)]. Thus, we reasoned that TMEM106B may modify disease risk by affecting DPR protein levels. To establish if loss of TMEM106B affects DPR protein levels, we introduced plasmids expressing transcripts containing 45 uninterrupted sense G4C2 repeats (G4C2 × 45) and 43 uninterrupted antisense CCCCGG repeats (C4G2 × 43) with V5 tags in all three frames downstream of the repeats (Supplementary Figures 1, 2) into HeLa cells that were treated with TMEM106B siRNA (siTMEM) or a non-targeting siRNA control (siCtrl). Similar to the C9orf72 repeat plasmids that we engineered without V5 tags (Hautbergue et al., 2017), these C9orf72 repeat constructs generate all five types of DPR proteins by RAN translation (Supplementary Figure 3) since they do not contain canonical AUG codons (Supplementary Figures 1, 2). TMEM106B expression was efficiently reduced by the siTMEM knockdown treatment (Figure 1A; source data is shown in Supplementary Figure 11). Using an anti-V5 antibody to detect the RAN-translated DPR proteins we found that knockdown of TMEM106B significantly increased the levels of both sense and antisense repeat-derived DPR proteins (Figure 1A). To further elaborate this result, we probed the samples with specific antibodies to poly(GA), poly(GR), poly(GP), and poly(PR) DPRs. Each of the expected DPR species translated from the sense and antisense repeats were detected and were increased in the siTMEM-treated samples (Figure 1B; source data is shown in Supplementary Figure 12).

**FIGURE 2:**
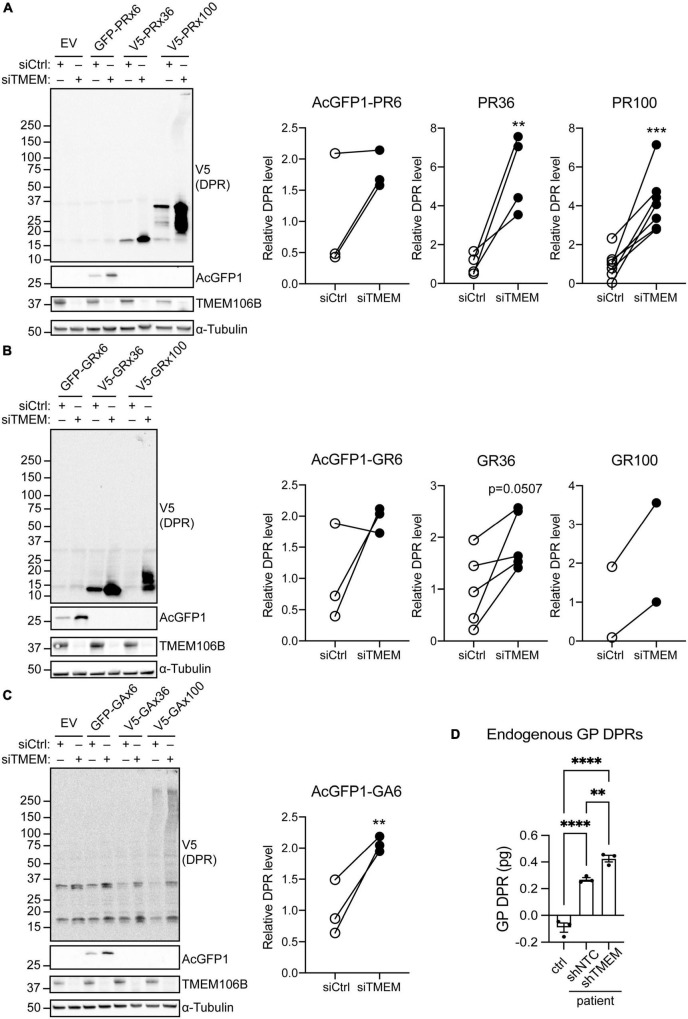
Knockdown of TMEM106B leads to accumulation of exogenous and endogenous C9ALS/FTD DPR proteins. **(A–C)** HeLa cells treated with either siCtrl or siTMEM were transfected with empty vector (EV) or AUG-driven synthetic, codon-optimized, AcGFP1-tagged 6 repeat, or V5-tagged 36 or 100 repeat poly(PR) **(A)**, poly(GR) **(B)** or poly(GA) **(C)** DPR constructs. Levels of AcGFP1- or V5-tagged DPRs and TMEM106B were determined on immunoblot. α-Tubulin indicates equal loading of samples. DPR levels were normalized to α-Tubulin and presented relative to siCtrl (unpaired *t*-test: **P ≤ 0.01, ***P ≤ 0.001; AcGFP1-PR6 *N* = 3, V5-PR36 *N* = 4, V5-PR100 *N* = 7, AcGFP1-GR6 *N* = 3, V5-GR36 *N* = 5, V5-GR100 *N* = 2, AcGFP1-GA6 *N* = 3 experiments). **(D)** Endogenous GP DPR levels were determined by MSD ELISA in C9ALS/FTD iAstrocytes (patient) transduced with non-targeting control (shNTC) or TMEM106b-specific shRNA (shTMEM) and iAstrocytes derived from a neurologically healthy control (ctrl) (mean ± SEM; one-way ANOVA with Fisher’s LSD test: **P ≤ 0.01, ****P ≤ 0.0001; *N* = 3).

To distinguish between an effect of TMEM106B on RAN translation efficiency or on clearance of DPR proteins we repeated the experiment using AUG-driven synthetic, codon-optimized, AcGFP1-tagged 6 repeat, or V5-tagged 36 and 100 repeat poly(PR), poly(GR) or poly(GA) DPR constructs (Mizielinska et al., 2014). All poly(PR) and poly(GR) DPR proteins were readily detected on immunoblots and showed markedly increased levels in the siTMEM-treated samples (Figures 2A,B; source data is shown in Supplementary Figure 13). Similarly, AcGFP1-tagged 6 repeat poly(GA) DPR protein levels were increased in TMEM106B knockdown samples. We were unable to reliably detect the 36 and 100 repeat poly(GA) DPR proteins in this assay (Figure 2C; source data is shown in Supplementary Figure 13).

To exclude effects of exogenous expression of the DPR constructs we turned to C9ALS/FTD patient-derived iAstrocytes with an endogenous G4C2 repeat expansion and a matched neurologically healthy control. MSD ELISA quantification of GP DPRs confirmed that compared to the control, C9ALS/FTD iAstrocytes contained detectable amounts of GP DPRs. Knockdown of TMEM106B using lentiviral shRNA significantly increased the level of GP DPRs in the C9ALS/FTD iAstrocytes (Figure 2D). Taken together, these data show that knockdown of TMEM106B causes accumulation of C9ALS/FTD-associated DPR proteins, which occurs independently of RAN translation efficiency.

### TMEM106B regulates autophagosome maturation

**FIGURE 3:**
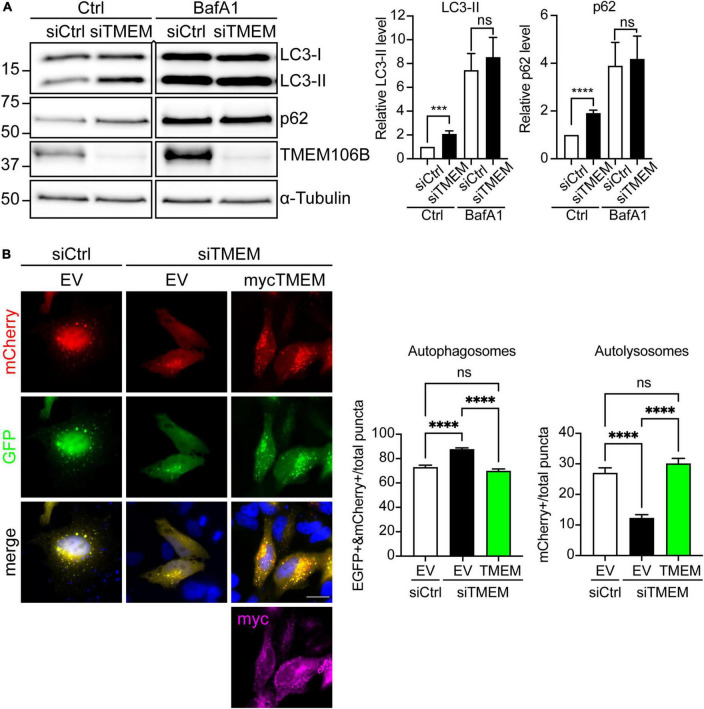
Loss of TMEM106B inhibits autophagy. **(A)** HeLa cells were treated with non-targeting control (siCtrl) or TMEM106B-targeted siRNA (siTMEM) for 4 days after which they were left untreated (Ctrl) or were treated with 100 nM Bafilomycin A1 (BafA1) for 6 h. The levels of LC3-II and SQSTM1/p62 were determined on immunoblot. α-Tubulin indicates equal loading of samples. LC3-II and SQSTM1/p62 levels were normalized against α-Tubulin and are presented relative to the siCtrl/Ctrl sample (mean ± SEM; unpaired *t*-test: ***P ≤ 0.001; ****P < 0.0001; siCtrl/Ctrl, siTMEM/Ctrl, *N* = 8; siCtrl/BafA1, siTMEM/BafA1, *N* = 7 experiments). An irrelevant lane was removed between the siTMEM/Ctrl and siCtrl/BafA1 lanes. **(B)** HeLa cells were treated with non-targeting control (siCtrl) or TMEM106B-targeted siRNA (siTMEM) for 3 days before transfection with mCherry-EGFP-LC3b and empty vector (EV) or myc-TMEM106B/T185. 24 h after transfection the cells were fixed and the number of EGFP/mCherry-positive (EGFP + &mCherry +) autophagosomes and mCherry-only-positive (mCherry +) autolysosomes quantified. Counts are presented relative to the combined count of autophagosomes and autolysosomes (mean ± SEM; one-way ANOVA with Fisher’s LSD test: ns not significant, ****P ≤ 0.0001; *N* = 92 (EV), 156 (siTMEM/EV), 93 (siTMEM/TMEM) cells from 3 experiments). Knockdown efficiency is shown in Supplementary Figure 4.

Since TMEM106B plays a role in lysosomal biology (Chen-Plotkin et al., 2012; Lang et al., 2012; Schwenk et al., 2014; Stagi et al., 2014; Jun et al., 2015; Busch et al., 2016; Kundu et al., 2018; Lüningschrör et al., 2020) and DPR proteins are autophagy substrates (Boivin et al., 2020), it seemed likely that the accumulation of DPR proteins may indicate impaired autophagy in TMEM106B-depleted cells. To test this directly we knocked down TMEM106B expression in HeLa cells using siRNA and monitored microtubule-associated protein 1A/1B light chain 3 (LC3) flux and SQSTM1/p62 levels (Figure 3). During autophagy, the cytosolic form of LC3 (LC3-I) is lipidated to form LC3-II. LC3-II, once recruited to autophagosomal membranes, remains membrane-associated throughout autophagy, until it is finally degraded in autolysosomes. Accordingly, the progression of autophagy is reflected in the turnover of LC3-II [Reviewed in Klionsky et al. (2021)]. SQSTM1/p62 levels serve as an index of autophagic degradation because as an autophagy receptor for ubiquitinated protein aggregates, it becomes incorporated in autophagosomes and is degraded alongside its cargo in autolysosomes. Hence increased levels of SQSTM1/p62 correlate with autophagy inhibition while a decrease is observed during activation of autophagy [Reviewed in Klionsky et al. (2021)].

Both LC3-II and SQSTM1/p62 levels were increased in siTMEM-treated samples compared to siCtrl-treated samples (Figure 3A). To further confirm whether this increase was due to a block in autophagy we used Bafilomycin A1, a V-ATPase inhibitor that causes an increase in lysosomal pH and blocks autophagosome maturation into autolysosomes. Under Bafilomycin A1 conditions, any increase in LC3-II levels specifically reflects enhanced induction of autophagy as opposed to a block in autophagy [Reviewed in Klionsky et al. (2021)]. In comparison to untreated cells, treatment with Bafilomycin A1 caused an increase in LC3-II and SQSTM1/p62 consistent with lysosomal inhibition and the resulting decrease in breakdown of LC3-II and SQSTM1/p62 by autophagy. Depletion of TMEM106B levels using siTMEM did not cause a further increase in LC3-II or SQSTM1/p62 compared to siCtrl-treated cells following Bafilomycin A1 treatment (Figure 3A). Thus, these data indicate that loss of TMEM106B blocks a late step in autophagy.

To further confirm the role of TMEM106B in autophagy we turned to a “traffic light” autophagy assay using EGFP-mCherry-LC3 [Reviewed in Klionsky et al. (2021)]. EGFP-mCherry-LC3 allows distinction between autophagosomes and autolysosomes (= autophagosome fused with lysosome) because EGFP fluorescence is quenched by the low pH in autolysosomes whereas mCherry fluorescence is not affected. Thus, autophagosomes fluoresce both red and green, whereas autolysosomes appear only red. Knockdown of TMEM106B expression induced a significant reduction in mCherry-only autolysosomes and a concomitant increase in yellow autophagosomes, indicating that maturation of autophagosomes to autolysosomes was impaired. To exclude possible off-target effects of the siTMEM treatment, we reintroduced TMEM106B/T185 into siTMEM-treated HeLa cells. Re-expression of TMEM106B rescued the maturation of autophagosomes to autolysosomes, confirming specificity of the siTMEM treatment (Figure 3B).

Previous work from our lab and others has shown that C9orf72 haploinsufficiency impairs autophagy and results in accumulation of SQSTM1/p62 and DPR proteins similar to what we find here after knockdown of TMEM106B (Sellier et al., 2016; Webster et al., 2016; Boivin et al., 2020; Zhu et al., 2020). Since TMEM106B levels modify C9ALS/FTD, we verified if siTMEM affected C9orf72 levels on immunoblots. Compared to siCtrl-treated cells, C9orf72 levels were increased after siTMEM treatment (Supplementary Figure 5A). Together, these data confirm TMEM106B as a novel regulator of autophagosome maturation.

### Loss of TMEM106B impairs lysosomal acidification and reduces cathepsin activity

**FIGURE 4:**
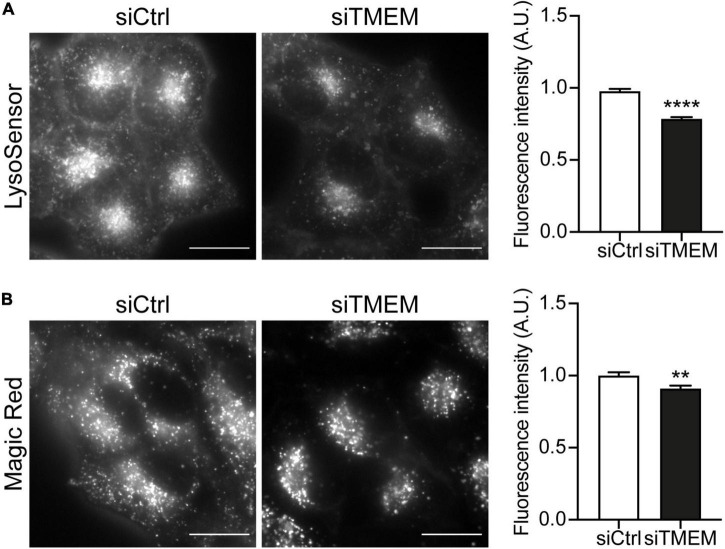
Knockdown of TMEM106B affects lysosomal pH and hydrolase activity. **(A)** Representative images of HeLa cells loaded with LysoSensor^®^ Green DND-189 following treatment with either siCtrl or siTMEM. Quantification of LysoSensor^®^ Green DND-189 fluorescence intensity per cell normalized to the mean intensity of siCtrl cells (mean ± SEM; unpaired *t*-test: ****P ≤ 0.0001; *N* = 4 experiments). **(B)** Representative images of cresyl violet fluorescence in HeLa cells incubated with cathepsin B Magic Red substrate following treatment with either siCtrl or siTMEM. Quantification of cresyl violet fluorescence intensity per cell normalized to the mean intensity of siCtrl cells (mean ± SEM; unpaired *t*-test: **P ≤ 0.01; *N* = 2 experiments). Knockdown efficiency is shown in Supplementary Figure 4.

Maturation of autophagosomes to autolysosomes is a complex dynamic process that requires sequential acquisition of lysosome membrane proteins and lysosomal hydrolases and acidification of the lumen. TMEM106B has been shown to regulate various aspects of lysosomal biology, including lysosomal acidification, hydrolase activity, and trafficking (Chen-Plotkin et al., 2012; Schwenk et al., 2014; Stagi et al., 2014; Busch et al., 2016; Klein et al., 2017; Kundu et al., 2018; Lüningschrör et al., 2020). Our novel data show that TMEM106B is involved in autophagosome maturation and regulates the clearance of DPR proteins by autophagy (Figures 1-3). To investigate if the effect of TMEM106B knockdown on autophagy involves changes to lysosomal acidification, we treated HeLa cells with either control or TMEM106B siRNA and measured lysosomal pH using LysoSensor^®^ Green DND-189, a fluorescent pH indicator that partitions into acidic organelles. LysoSensor^®^ Green DND-189 is almost non-fluorescent except when inside an acidic compartment. Thus, LysoSensor^®^ Green DND-189 fluorescence intensity inversely correlates with pH. Knockdown of TMEM106B expression caused a decrease of approximately 20% in LysoSensor^®^ Green DND-189 fluorescence, consistent with reduced acidification of lysosomes (Figure 4A and Supplementary Figure 5A). Lysosomal pH is known to regulate hydrolase activity and as a consequence the degradative capacity of lysosomes. To assess the effect of the increase in lysosomal pH caused by TMEM106B knockdown on the degradative capacity of lysosomes we measured the activity of cathepsin B using a Magic Red^®^ Cathepsin B assay. Hydrolysis of the cathepsin B Magic Red^®^ substrate by catalytically active cathepsin B in lysosomes releases membrane-impermeable fluorescent cresyl violet within lysosomes and allows easy quantification of cathepsin B activity by fluorescence microscopy (Creasy et al., 2007; Bright et al., 2016). Knockdown of TMEM106B decreased the activity of cathepsin B only marginally (< 10%), indicating that despite the increase in pH measured with LysoSensor^®^, the degradative capacity of lysosomes was largely intact (Figure 4B and Supplementary Figure 5B). Thus, knockdown of TMEM106B caused only a moderate reduction in lysosome acidification and cathepsin B activity.

### TMEM106B regulates Arl8b-mediated trafficking of lysosomes to the cell periphery

Autophagosome/lysosome fusion is modulated by bidirectional movement of autophagosomes and lysosomes [Reviewed in Jia et al. (2017), Cabukusta and Neefjes (2018), Ballabio and Bonifacino (2020)], and it has been reported that TMEM106B levels affect lysosomal trafficking (Schwenk et al., 2014; Stagi et al., 2014; Lüningschrör et al., 2020). Therefore, since knockdown of TMEM106B had only modest effects on lysosomal acidification and enzyme activity, we reasoned that the impairment of autophagosome maturation after TMEM106B knockdown may be related to disrupted lysosomal trafficking.

**FIGURE 5:**
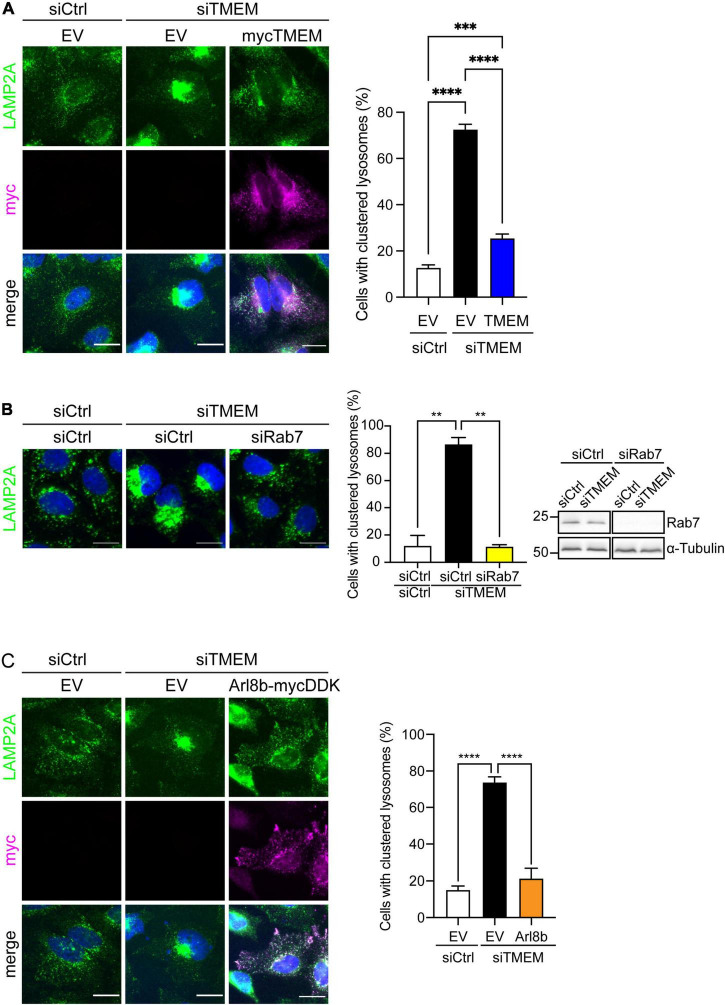
TMEM106B regulates Arl8b-mediated trafficking of lysosomes to the cell periphery. **(A)** HeLa cells were treated with either siCtrl or siTMEM and transfected as indicated with empty vector (EV) or myc-tagged TMEM106B/T185. Cells were immunostained for the endogenous lysosomal marker LAMP2A (green) and transfected myc-TMEM106B (magenta). Images were blinded and lysosomes were classified as clustered or dispersed based on the distribution of LAMP2A. The percentage of cells with clustered lysosomes in each condition are presented (mean ± SEM; one-way ANOVA with Fisher’s LSD test: ***P ≤ 0.001, ****P ≤ 0.0001; siCtrl/EV and siTMEM/EV, *N* = 7 experiments; siTMEM/TMEM, *N* = 6 experiments. Knockdown efficiency is shown in Supplementary Figure 4. **(B)** HeLa cells were treated with either siCtrl, siTMEM, or siTMEM together with a pool of Rab7A targeted siRNA (siRab7) and immunostained for endogenous LAMP2A (green). Images were blinded and lysosomes classified as clustered or dispersed based on the distribution of LAMP2A. The percentage of cells with clustered lysosomes in each condition are presented (mean ± SEM; one-way ANOVA with Fisher’s LSD test: **P ≤ 0.01; *N* = 2 experiments. Scale bar = 20 μm. Knockdown efficiency was determined by immunoblot of Rab7 using tubulin as loading control. **(C)** HeLa cells treated with either siCtrl or siTMEM and transfected as indicated with empty vector (EV) or mycDDK-tagged Arl8b (Arl8b-mycDDK) were immunostained for the endogenous lysosomal marker LAMP2A (green) and transfected Arl8b-mycDDK (magenta). Images were blinded and lysosomes were classified as clustered or dispersed based on the distribution of LAMP2A. The percentage of cells with clustered lysosomes in each condition are presented (mean ± SEM; one-way ANOVA with Fisher’s LSD test: ****P ≤ 0.0001; *N* = 3 experiments. Scale bar = 20 μm. Knockdown efficiency is shown in Supplementary Figure 4.

We first analyzed the effect of TMEM106B knockdown on lysosomal motility in HeLa cells. We treated HeLa cells with siCtrl or siTMEM and determined the distribution of lysosomes using immunofluorescence microscopy of the endogenous lysosomal marker LAMP2A. To exclude possible effects of TMEM106B knockdown on LAMP2A levels we determined LAMP2A levels in siCtrl and siTMEM-treated samples on immunoblot. TMEM106B knockdown did not affect LAMP2A levels in these assays (Supplementary Figure 6). In siCtrl-treated HeLa cells lysosomes were distributed throughout the cell, with a characteristic population of perinuclear lysosomes and numerous peripheral lysosomes. In contrast siTMEM treatment caused a collapse of the lysosomal population to the juxtanuclear area of the cell, with the peripheral population of lysosomes largely absent and most, if not all, lysosomes present in a distinct juxtanuclear cluster (Figure 5A and Supplementary Figure 7A). To exclude possible off-target effects of the siTMEM treatment, we reintroduced TMEM106B/T185 into siTMEM-treated HeLa cells. TMEM106B/T185 localized to lysosomes and fully rescued lysosomal clustering, confirming specificity of the siTMEM treatment (Figure 5A). Thus, TMEM106B is required to maintain lysosomes in the cell periphery.

Juxtanuclear clustering of lysosomes indicates an imbalance between anterograde and retrograde transport in favor of retrograde transport. Retrograde transport of lysosomes toward the cell center is mediated by the small GTPase Rab7A. When activated in its GTP-bound state lysosome-associated Rab7A recruits its effector protein Rab-interacting lysosomal protein (RILP), which in turn recruits the cytoplasmic dynein motor that drives retrograde transport. Multiple mechanisms of anterograde transport of lysosomes toward the cell periphery have been identified. One of these is also Rab7A mediated; in addition to RILP, activated Rab7A can bind the effector FYCO1 which in combination with phosphatidylinositol 3-phosphate (PI(3)P) recruits the kinesin-1 motor which drives anterograde transport. An alternative mechanism involves the small GTPase ADP-ribosylation factor (Arf)-like protein 8b (Arl8b), which upon activation recruits the effector SKIP to engage in kinesin-1 driven anterograde transport [Reviewed in Cabukusta and Neefjes (2018)].

We first investigated the involvement of Rab7A by double knockdown of Rab7A and TMEM106B in HeLa cells. Depletion of Rab7A using siRNA rescued lysosomal clustering caused by knockdown of TMEM106B (Figure 5B and Supplementary Figure 7B). Hence, Rab7A-mediated retrograde transport activity is intact in the absence of TMEM106B and drives juxtanuclear clustering of lysosomes. Since knockdown of Rab7A restored the balance between retrograde and anterograde trafficking of lysosomes, these data pointed toward Arl8b as the main driver of anterograde transport of lysosomes in these cells, and suggested that knockdown of TMEM106B may impair Arl8b-mediated anterograde transport. To test this hypothesis, we overexpressed Arl8b in siTMEM-treated cells and analyzed the distribution of lysosomes. Arl8b overexpression completely rescued the lysosomal clustering phenotype in siTMEM-treated cells (Figure 5C and Supplementary Figure 7C).

One possibility was that TMEM106B knockdown might affect Rab7A and/or Arl8b levels to alter the balance between retrograde and anterograde lysosomal trafficking. However, when we analyzed Arl8b levels on immunoblots we found that Arl8b levels were increased in cells with reduced TMEM106B expression compared to control siRNA treated cells, while Rab7A expression was unchanged (Supplementary Figure 8). Thus, these data are consistent with a model in which Arl8b-mediated anterograde transport is reduced after TMEM106B knockdown, thereby preferentially facilitating Rab7A-mediated retrograde transport, and resulting in juxtanuclear lysosomal clustering.

### Arl8b rescues impaired autophagy and C9ALS/FTD DPR levels in TMEM106B-depleted cells

**FIGURE 6:**
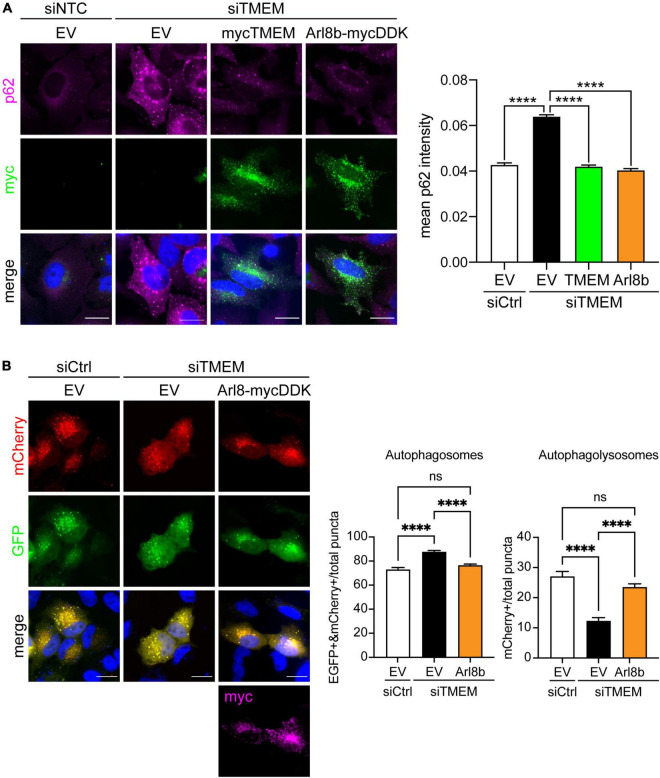
Restoring Arl8b-mediated lysosomal trafficking rescues impaired autophagy in TMEM106B-depleted cells. **(A)** HeLa cells treated with either siCtrl or siTMEM106B were transfected with empty vector (EV), myc-tagged TMEM106B/T185, or mycDDK-tagged Arl8b (Arl8b-mycDDK). Cells were immunostained for endogenous SQSTM1/p62 (magenta) and the transfected myc-tag (green). Accumulation of SQSTM1/p62 was quantified as the mean intensity per cell (mean ± SEM; one-way ANOVA with Fisher’s LSD test, ****P ≤ 0.0001; *N* = 907 (siCtrl/EV), 1,488 (siTMEM/EV), 456 (siTMEM/TMEM), 367 (siTMEM/Arl8b) cells from 3 experiments). Scale bar = 20 μm. **(B)** HeLa cells were treated with non-targeting control (siCtrl) or TMEM106B targeted siRNA (siTMEM) for 3 days before transfection with mCherry-EGFP-LC3b and empty vector (EV) or Arl8b-mycDDK. 24 h after transfection the cells were fixed and the number of EGFP/mCherry-positive autophagosomes and mCherry-only-positive autolysosomes quantified. Counts are presented relative to the combined count of autophagosomes and autolysosomes (mean ± SEM; one-way ANOVA with Fisher’s LSD test: ns not significant, ****P ≤ 0.0001; *N* = 92 (siCtrl/EV), 156 (siTMEM/EV), 166 (siTMEM/Arl8b) cells from 3 experiments; siCtrl/EV and siTMEM/EV are identical to Figure 3B). Knockdown efficiency is shown in Supplementary Figure 4.

Several lines of research show that autophagosome maturation requires the correct trafficking of lysosomes. Autophagosomes move from the cell periphery to the perinuclear area by retrograde transport to fuse with juxtanuclear lysosomes. Conversely lysosome dispersal increases the fusion of lysosomes with peripheral autophagosomes [Reviewed in Jia et al. (2017), Cabukusta and Neefjes (2018), Zhao and Zhang (2019), Ballabio and Bonifacino (2020)]. Accordingly, we reasoned that the anterograde lysosomal trafficking deficit caused by TMEM106B knockdown could be the underlying cause of the autophagy impairment we observed (Figure 3). To test this, we restored anterograde lysosomal trafficking in TMEM106B knockdown cells by expression of Arl8b and measured autophagy by determining SQSTM1/p62 levels (Figure 6A and Supplementary Figure 9A). In these experiments we visualized SQSTM1/p62 by immunofluorescence microscopy rather than on immunoblots to allow specific analysis of Arl8b-transfected cells. In agreement with the immunoblot data in Figure 3, immunofluorescence analysis showed that knockdown of TMEM106B caused a marked increase in SQSTM1/p62 which could be completely rescued by reintroducing TMEM106B. Overexpression of Arl8b in siTMEM-treated cells, which restored lysosome distribution (Figure 5C), reduced SQSTM1/p62 fluorescence intensity to control levels (Figure 6A). To further confirm this result, we again used the EGFP-mCherry-LC3 “traffic light” assay to analyze autophagy flux [Reviewed in Klionsky et al. (2021)]. The number of autophagosomes and autolysosomes was restored to control levels in siTMEM-treated cells upon expression of Arl8b (Figure 6B and Supplementary Figure 9B). Thus, reinstating lysosomes in the cell periphery restores autophagy in TMEM106B-depleted cells.

**FIGURE 7:**
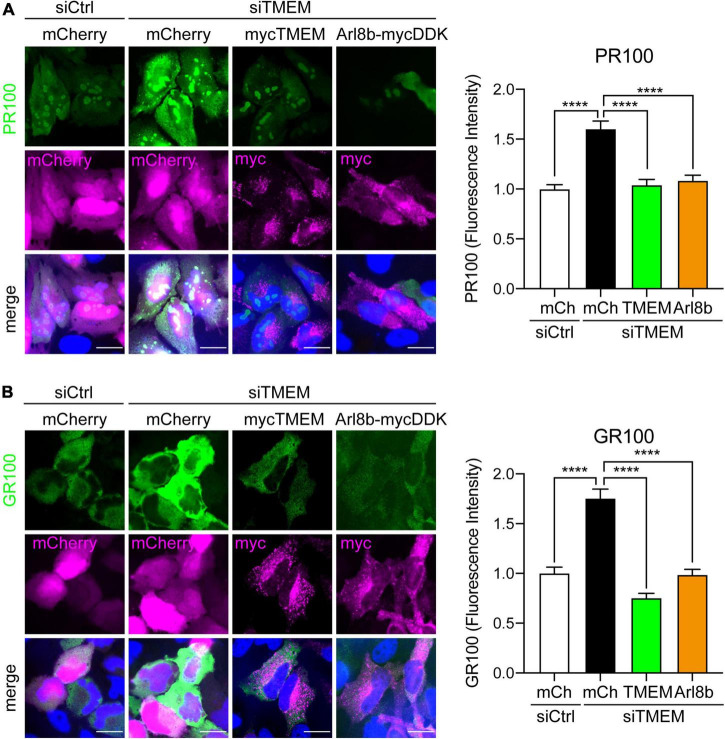
Restoring Arl8b-mediated lysosomal trafficking rescues C9ALS/FTD DPR levels in TMEM106B-depleted cells. HeLa cells treated with either siCtrl or siTMEM106B were transfected with mCherry, myc-tagged TMEM106B/T185, or mycDDK-tagged Arl8b (Arl8b-mycDDK) together with control mCherry (mCh), V5-tagged 100 repeat poly(PR) (PR100) **(A)** or V5-tagged 100 repeat poly(GR) (GR100) **(B)**. Cells were immunostained for V5 (green) and myc (magenta); mCherry fluorescence (magenta) was visualized when present. The mean V5-DPR intensity per cell was quantified for each condition and normalized to the mean intensity in siCtrl-treated cells (mean ± SEM; one-way ANOVA with Fisher’s LSD test, ****P ≤ 0.0001; **(A)** *N* = 419 (siCtrl/mCh), 448 (siTMEM/mCh), 496 (siTMEM/TMEM), 473 (siTMEM/Arl8b), **(B)** *N* = 341 (siCtrl/mCh), 354 (siTMEM/mCh), 350 (siTMEM/TMEM), 326 (siTMEM/Arl8b) cells from 3 experiments). Scale bar = 20 μm. Knockdown efficiency is shown in Supplementary Figure 4.

Since TMEM106B depletion blocks autophagy and causes accumulation of C9ALS/FTD DPR proteins, we next enquired if rescuing autophagy by restoring anterograde lysosomal trafficking affected DPR protein levels. Again, we utilized fluorescence microscopy for these assays to allow specific investigation of transfected cells. Similar to the immunoblot data in Figure 2, siTMEM-treated cells showed increased levels of synthetic 100 repeat poly(PR) or poly(GR) DPR protein compared to siCtrl-treated cells. This was specific for loss of TMEM106B expression because co-transfected TMEM106B/T185 fully rescued the increased accumulation of DPR proteins in siTMEM-treated cells (Figure 7 and Supplementary Figure 10). Similarly, co-expression of Arl8b, which we have shown to rescue lysosomal distribution and autophagy (Figure 6), reduced the level of DPR proteins to control levels (Figure 7). Thus, we have demonstrated that loss of TMEM106B causes an imbalance in bidirectional lysosome trafficking, which impairs autophagy and consequently leads to increased C9ALS/FTD DPR protein accumulation.

## Discussion

This study shows that reduction of TMEM106B expression impairs autophagy (Figures 3, 6) and exacerbates exogenous and endogenous DPR protein pathology (Figures 1, 2, 7), suggesting the possibility that the disease modifying effect of *TMEM106B* SNPs in C9orf72 repeat expansion carriers is driven by changes in DPR levels. Indeed, an increase in DPR-mediated toxicity may explain why reduced levels of TMEM106B, while protective of developing FTLD, associate with earlier onset of disease and death in C9orf72 expansion carriers (Gallagher et al., 2014). By analogy to TMEM106B it has been shown by several groups, including our own, that C9orf72 regulates autophagy (Amick et al., 2016; Sellier et al., 2016; Sullivan et al., 2016; Webster et al., 2016; Yang et al., 2016), and at least in cultured cells this deficit in autophagy leads to increased DPR protein pathology (Boivin et al., 2020). Thus, autophagy emerges as a modifier of C9ALS/FTD by modulation of DPR protein toxicity, and autophagy is in turn regulated by both TMEM106B and C9orf72 expression levels.

In agreement with this model, inactivation of one or both endogenous *C9orf72* alleles exacerbated DPR protein accumulation and the neurodegenerative phenotype in mice expressing 66 G4C2 repeats after AAV injection and in a 450 repeat *C9orf72* BAC transgenic mouse model (Zhu et al., 2020). In contrast to our data and the modifying effects of C9orf72 expression levels, there was no increase in soluble poly(GP) levels in the brains of TMEM106B knockout mice expressing 66 G4C2 repeats after AAV injection compared to wild type brains (Nicholson et al., 2018). Similar to our findings here, a defect in lysosome trafficking and impairment of autophagy has been reported in a TMEM106B knockout mouse model generated using a CRISPR/Cas9 strategy (Lüningschrör et al., 2020). However, this phenotype was not reported in the TMEM106B knockout mice generated using a gene-trap strategy that were used to investigate TMEM106B in C9ALS/FTD (Klein et al., 2017; Nicholson et al., 2018). Gene-trap TMEM106B knockout mice contain a residual N-terminal fragment of TMEM106B (Nicholson et al., 2018) and it has been suggested that the gene-trap strategy may lead to incomplete knockout of TMEM106B (Lüningschrör et al., 2020). It will be important to thoroughly characterize the different TMEM106B knockout models to allow robust evaluation of the *in vivo* effects of TMEM106B levels in C9ALS/FTD as well as progranulin FTD mouse models.

We show that TMEM106B regulates bidirectional trafficking of lysosomes. Knockdown of TMEM106B caused a deficit in anterograde, Arl8b-mediated transport, which shifted the balance of bidirectional transport toward Rab7A-mediated retrograde transport and caused distinct juxtanuclear clustering of lysosomes (Figure 5). These data are consistent with observations in neuronal cultures that show increased retrograde transport of Rab7A positive lysosomes, reduced numbers of lysosomes in processes, and accumulation of lysosomes in the initial segment of axons after knockdown or knockout of TMEM106B (Schwenk et al., 2014; Lüningschrör et al., 2020) indicating that the underlying mechanisms are shared between neuronal and non-neuronal cell types. In agreement with our data, clustering of lysosomes was also reported in CRISPR/Cas9 TMEM106B knockout HeLa and primary oligodendrocytes derived from TMEM106B knockout mice (Zhou et al., 2020).

Lysosomal motility has been linked to fusion of lysosomes with late endosomes and autophagosomes, but the exact role of lysosomal motility in fusion remains elusive. Both Rab7A and Arl8b have been shown to also play a role in fusion of lysosomes with late endosomes and autophagosomes by recruiting PLEKHM1 and subunits of the mammalian homotypic fusion and vacuole protein sorting (HOPS) complex to their target membranes (Khatter et al., 2015; Jia et al., 2017; Marwaha et al., 2017; Boda et al., 2019). In turn, PLEKHM1 and HOPS act as tethering/scaffold factors to bring the two membranes in close proximity and to enable SNARE complex assembly and ultimately membrane fusion [Reviewed in Ballabio and Bonifacino (2020)]. Interestingly a yeast 2-hybrid screen using the cytoplasmic domain of TMEM106B showed a positive interaction with the HOPS complex subunit Vps11 (Stagi et al., 2014). The interaction of TMEM106B and Vps11 remains to be confirmed in mammalian cells, but if confirmed, it suggests that TMEM106B may also regulate lysosomal fusion events.

Lysosome motility has been shown to be critical for many lysosomal functions including autophagy [Reviewed in Ballabio and Bonifacino (2020); Reviewed in Cabukusta and Neefjes (2018)]. Specifically, it has been suggested that impaired movement of lysosomes to the periphery and the resulting clustering of lysosomes inhibits autophagy because of reduced encounters between lysosomes and autophagosomes in the peripheral cytoplasm, where many autophagosomes are formed (Jia et al., 2017). Accordingly, we found that the impairment of autophagy in TMEM106B knockdown cells could be rescued by restoring the pool of peripheral lysosomes using Arl8b (Figure 6). Importantly, this also reduced DPR protein accumulation in cells lacking TMEM106B (Figure 7), indicating a possible novel therapeutic avenue for C9ALS/FTD.

There is a strong link between autophagy/lysosomal dysfunction and ALS/FTD. Mutations in genes known to function in autophagy and/or lysosomes directly (SQSTM1/p62, TBK1, OPTN, Ubiquilin-2), or indirectly (C9orf72, VCP, CHMP2B, VAPB, ALS2, and DCTN1) cause familial forms of the disease, while the near universal occurrence of aggregated protein pathology argues for a role of defects in autophagy and/or lysosomal function regardless of the underlying cause of disease [Reviewed in Webster et al. (2017)]. We now show that TMEM106B is required for autophagy and functions at a late stage in the autophagy pathway (Figure 3), further strengthening this link. Crucially we show that restoring autophagy, in this case by correcting the lysosomal positioning phenotype, ameliorated C9ALS/FTD DPR protein accumulation in TMEM106B knockdown cells (Figures 6, 7). Thus, autophagy enhancing treatments may be of benefit for C9ALS/FTD and require further investigation. At the same time, our data raise concern in relation to therapeutic strategies in FTD based on lowering TMEM106B levels, especially in the case of C9ALS/FTD. Indeed, our data indicate that lowering TMEM106B levels, while possibly decreasing the odds of developing FTLD, may exacerbate the disease by increasing DPR protein accumulation.

Finally, variants in TMEM106B have been shown to be associated with risk in several aging-related diseases in addition to ALS/FTD, including Parkinson’s disease, Alzheimer’s disease, and limbic-predominant age related TDP-43 encephalopathy (LATE) neuro-pathological change (LATE-NC) with or without coexisting hippocampal sclerosis pathology, and may in fact confer a neuronal protection effect against general aging independent of disease status (Satoh et al., 2014; Nelson et al., 2019; Tropea et al., 2019; Li et al., 2020). Since neuron-specific knockout of the essential autophagy genes ATG7, ATG5, and RB1CC1 (FIP200) in mice causes neurodegeneration (Hara et al., 2006; Komatsu et al., 2006; Liang et al., 2010) it will be of interest to explore if deficits in autophagy also explain the risk conferred by *TMEM106B* SNPs in these human conditions.

## Data availability statement

The raw data supporting the conclusions of this article will be made available by the authors, without undue reservation.

## Author contributions

CB, CW, and ES performed all experiments. AS, JK, and LF provided the iAstrocytes. CB, CW, and KDV analyzed the data, designed the experiments, and wrote the manuscript with input from all authors. FI-Á, LC, Y-HL, AH, and GH generated key reagents for the study. AH performed MSD assays. PS provided genetically characterized human fibroblasts for reprogramming. AG and KDV conceived the study. KDV supervised the research. All authors contributed to the article and approved the submitted version.
