# Supplementary material for: Loss of TMEM106B exacerbates C9ALS/FTD DPR pathology by disrupting autophagosome maturation
Source: Front Cell Neurosci. 2022 Dec 16;16:1061559. doi: 10.3389/fncel.2022.1061559 (PMC9812496; doi:10.3389/fncel.2022.1061559)
Supplement: Supplementary file 1 [file Data_Sheet_1.pdf]

Supplementary Figures

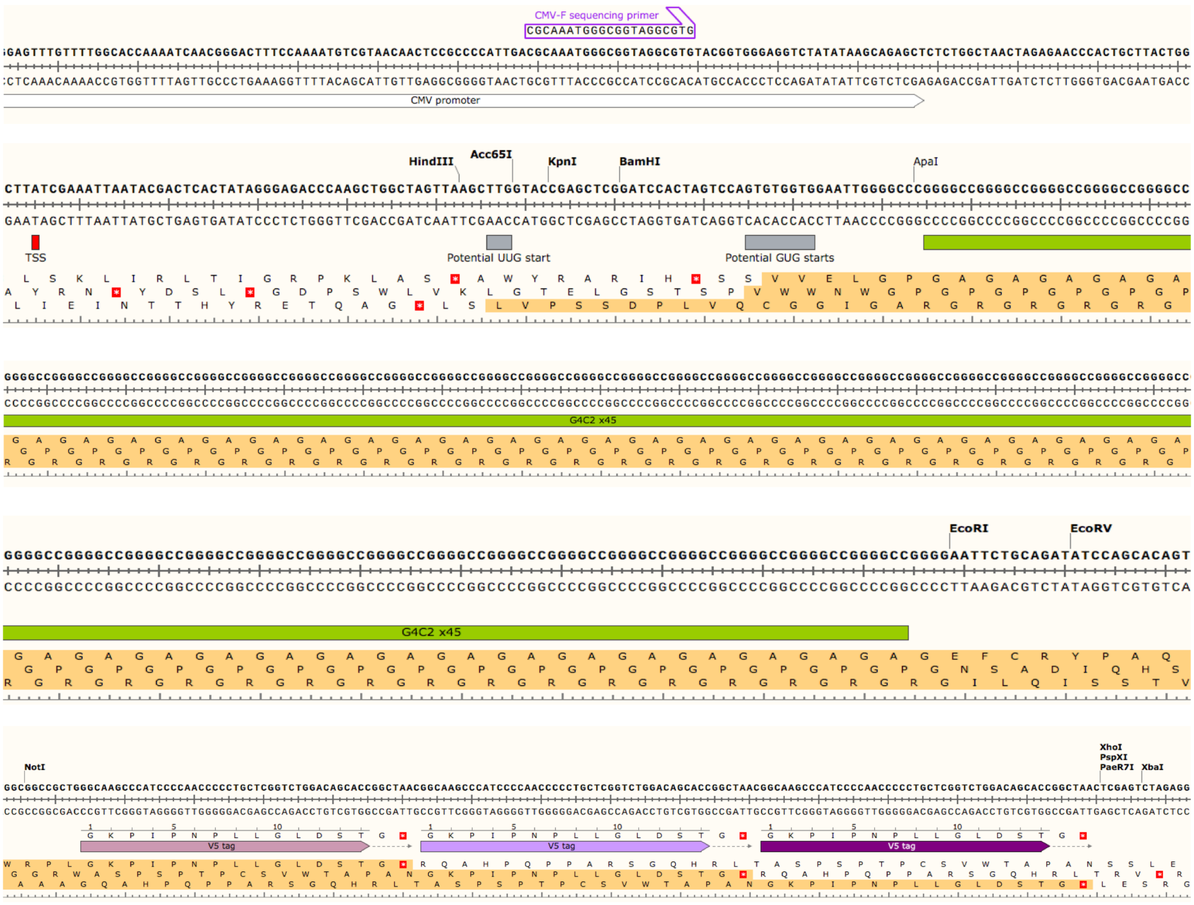

Supplementary Figure 1: Engineering RAN-dependent G4C2x45 repeat transcripts with 3xV5 tags in all open reading frames. Concatemerized and annealed G4C2/C4G2x15 DNA oligonucleotides blunted with Mung bean nuclease were cloned into the Klenow-filled site EcoRI of pcDNA3.1 to build pcDNA3.1-G4C2x45. A NotI/XbaI cassette encoding 3xV5 tags and 3 stop codons in all frames was digested from a synthetic custom-synthesised plasmid (ThermoFisher Scientific) and subcloned into the NotI/XbaI site of pcDNA3.1-G4C2x45. Sanger sequencing is available on request. Note the absence of canonical AUG start codons from the transcription start site (TSS) to investigate RAN-dependent translation of sense C9ORF72 repeat transcripts. The one letter amino-acid code sequences highlighted in orange indicate the DPRs respectively produced in the 3 reading frames.

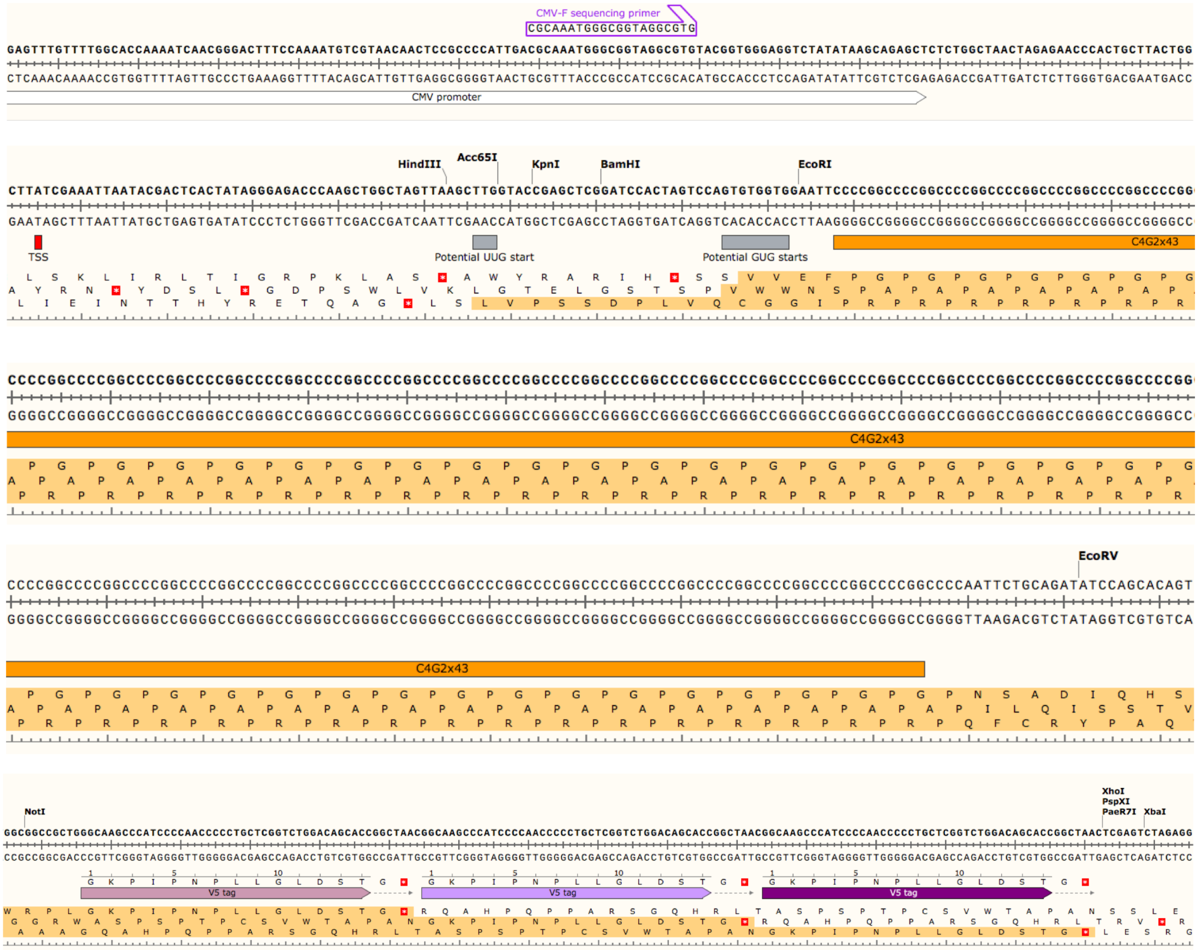

Supplementary Figure 2: Engineering RAN-dependent C4G2x43 repeat transcripts with 3xV5 tags in all open reading frames. Concatemerized and annealed G4C2/C4G2x15 DNA oligonucleotides blunted with Mung bean nuclease were cloned into the Klenow-filled site EcoRI of pcDNA3.1 to build pcDNA3.1-C4G2x43. A NotI/XbaI cassette encoding 3xV5 tags and 3 stop codons in all frames was digested from a synthetic custom-synthesized plasmid (ThermoFisher Scientific) and subcloned into the NotI/XbaI site of pcDNA3.1-C4G2x43. Sanger sequencing is available on request. Note the absence of canonical AUG start codons from the transcription start site (TSS) to investigate RAN-dependent translation of sense C9ORF72 repeat transcripts.The one letter amino-acid code sequences highlighted in orange indicate the DPRs respectively produced in the 3 reading frames.

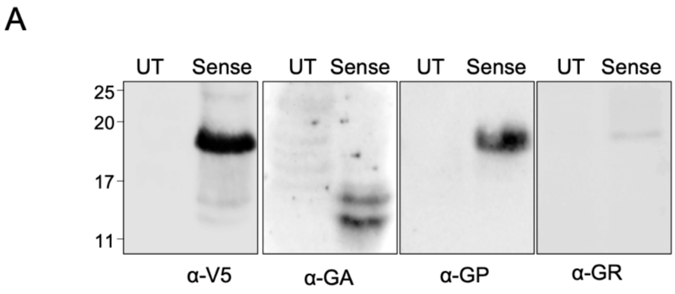

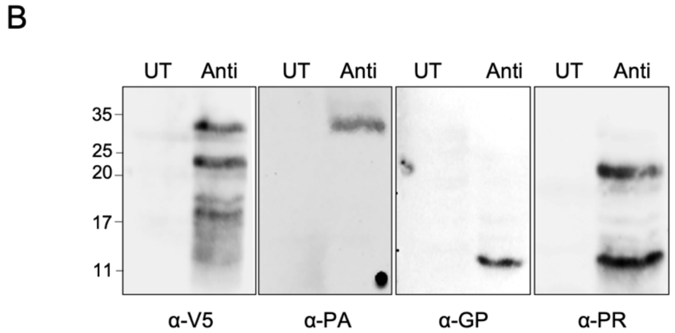

Supplementary Figure 3: RAN translation dependent sense and antisense reporter constructs produce all species of V5-tagged DPRs

(A) Protein extracts from HEK293T transfected for 48 hours with pcDNA3.1-G4C2x45-3xV5 were subjected to immunoblot analysis (described in Hautbergue *et al.*, 2017) using antibodies against V5 (Invitrogen R960), poly-GA (Proteintech 24492-1-AP), poly-GP (Proteintech 24494-1-AP) and poly-GR (Proteintech 23978-1-AP).

(B) Protein extracts from HEK293T transfected for 48 hours with pcDNA3.1-C4G2x43-3xV5 were subjected to western blot analysis using antibodies against V5 (Invitrogen R960), poly-PA (Merck MABN1790), poly-GP (Proteintech 24494-1-AP) and poly-PR (Proteintech 23979-1-AP).

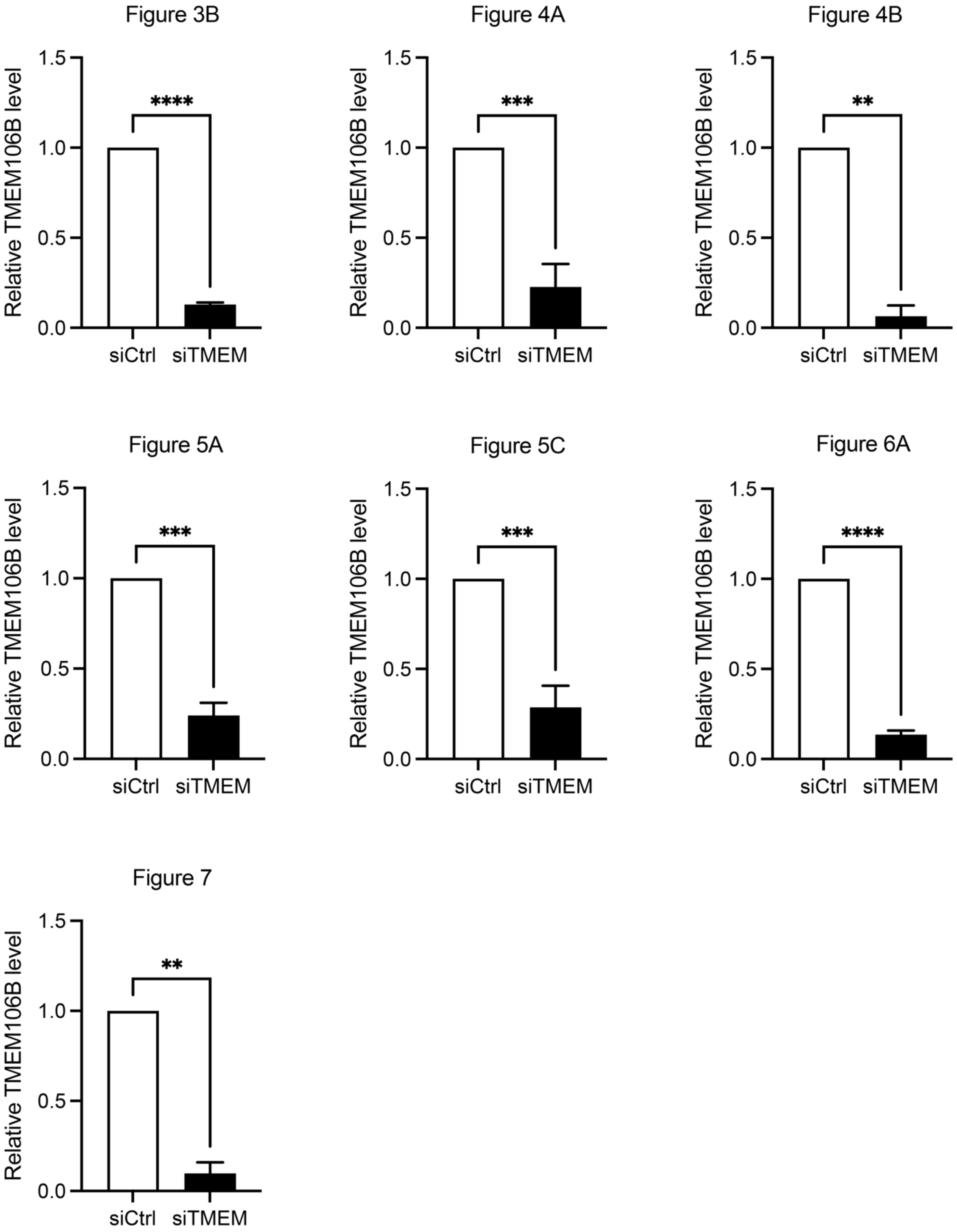

Supplementary Figure 4: Quantification of TMEM106B knockdown efficiency.
HeLa cells were treated with non-targeting control (siCtrl) or TMEM106B targeted siRNA (siTMEM) for 4 days. Cells were harvested and the levels of TMEM106B determined on immunoblot. Matching figures are indicated above each panel (mean ± SEM; unpaired t-test:

Fig 3B/6B: ****$P \leq 0.0001$, N = 3 experiments; Fig 4A: ***$P \leq 0.001$, N = 4 experiments; Fig4B: **$P \leq 0.01$, N = 2 experiments; Fig. 5A: ***$P \leq 0.001$, N = 3 experiments; Fig. 5C: ***$P \leq 0.001$, N = 4 experiments; Fig. 6A: ****$P \leq 0.0001$, N = 3 experiments; Fig. 7: **$P \leq 0.01$, N = 3 experiments).

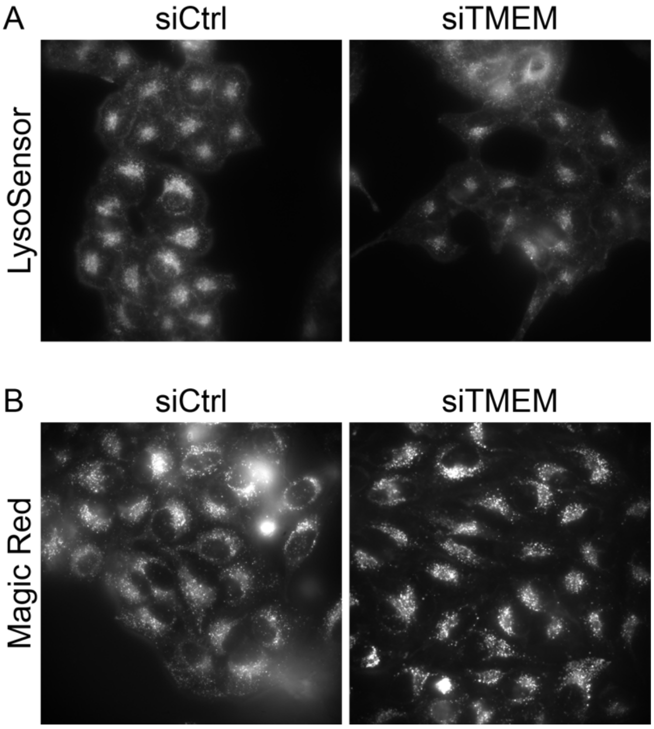

Supplementary Figure 5: Knockdown of TMEM106B affects lysosomal pH and hydrolase activity; full images of Figure 4. (A) Representative images of HeLa cells loaded with LysoSensor® Green DND-189 following treatment with either siCtrl or siTMEM106B. (B) Representative images of cresyl violet fluorescence in HeLa cells incubated with cathepsin B Magic Red substrate following treatment with either siCtrl or siTMEM106B.

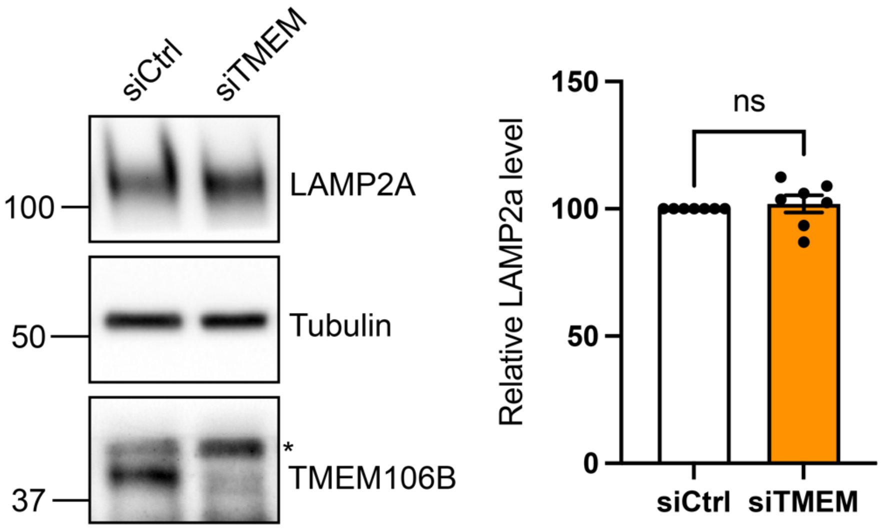

Supplementary Figure 6: TMEM106B knockdown does not affect LAMP2A levels

HeLa cells treated with either siCtrl or siTMEM106B were harvested and the level of TMEM106B, and LAMP2A determined on immunoblot. LAMP2A levels were normalised against α-Tubulin and presented relative to siCtrl (mean ± SEM; unpaired t-test: ns, not significant; N = 7 experiments). * denotes an non-specific band.

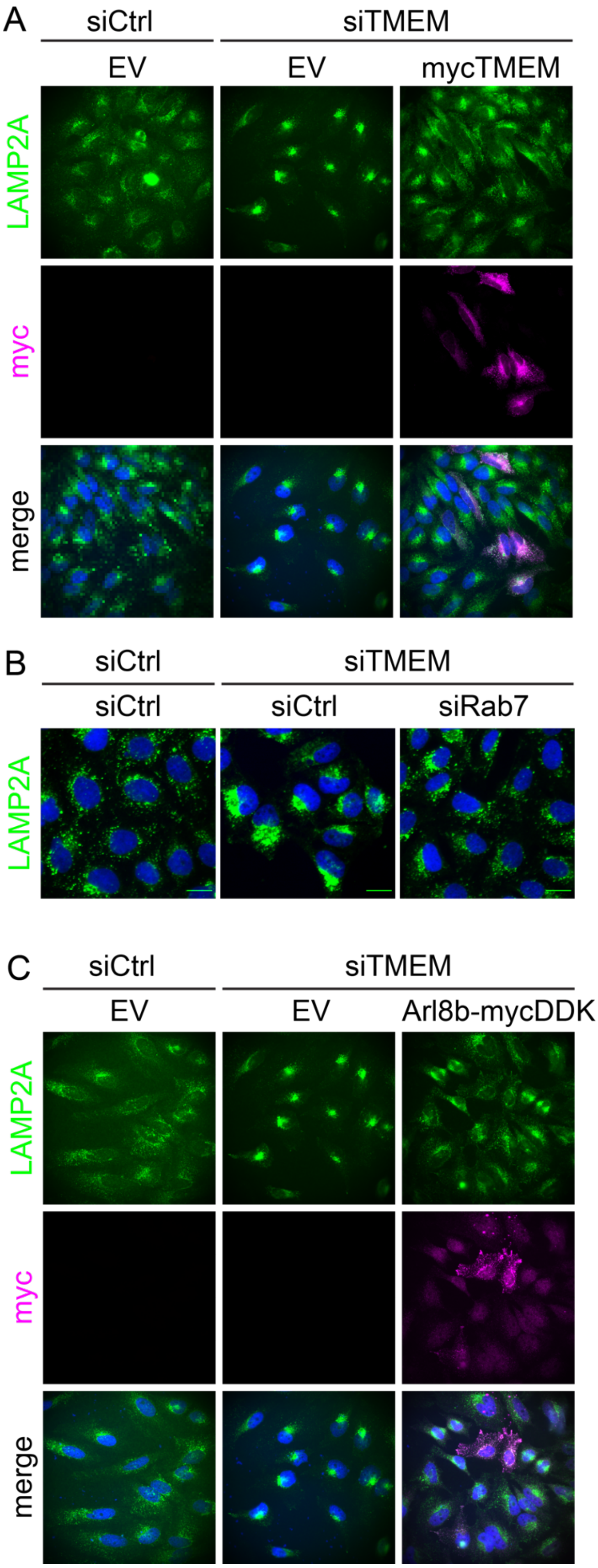

Supplementary Figure 7: TMEM106B regulates Arl8b-mediated trafficking of lysosomes to the cell periphery; full images of Figure 5. (A) HeLa cells were treated with either siCtrl

or siTMEM106B and transfected as indicated with empty vector (EV) or myc-tagged TMEM106B/T185. Cells were immunostained for the endogenous lysosomal marker LAMP2A (green) and transfected myc-TMEM106B (magenta).

(B) HeLa cells were treated with either siCtrl, siTMEM106B, or siTMEM106B together with a pool of Rab7A targeted siRNA (siRab7) and immunostained for endogenous LAMP2A (green).

(C) HeLa cells treated with either siCtrl or siTMEM106B and transfected as indicated with empty vector (EV) or mycDDK-tagged Arl8b (Arl8b-mycDDK) were immunostained for the endogenous lysosomal marker LAMP2A (green) and transfected myc-Arl8b (magenta). Images were blinded and lysosomes were classified as clustered or dispersed based on the distribution of LAMP2A.

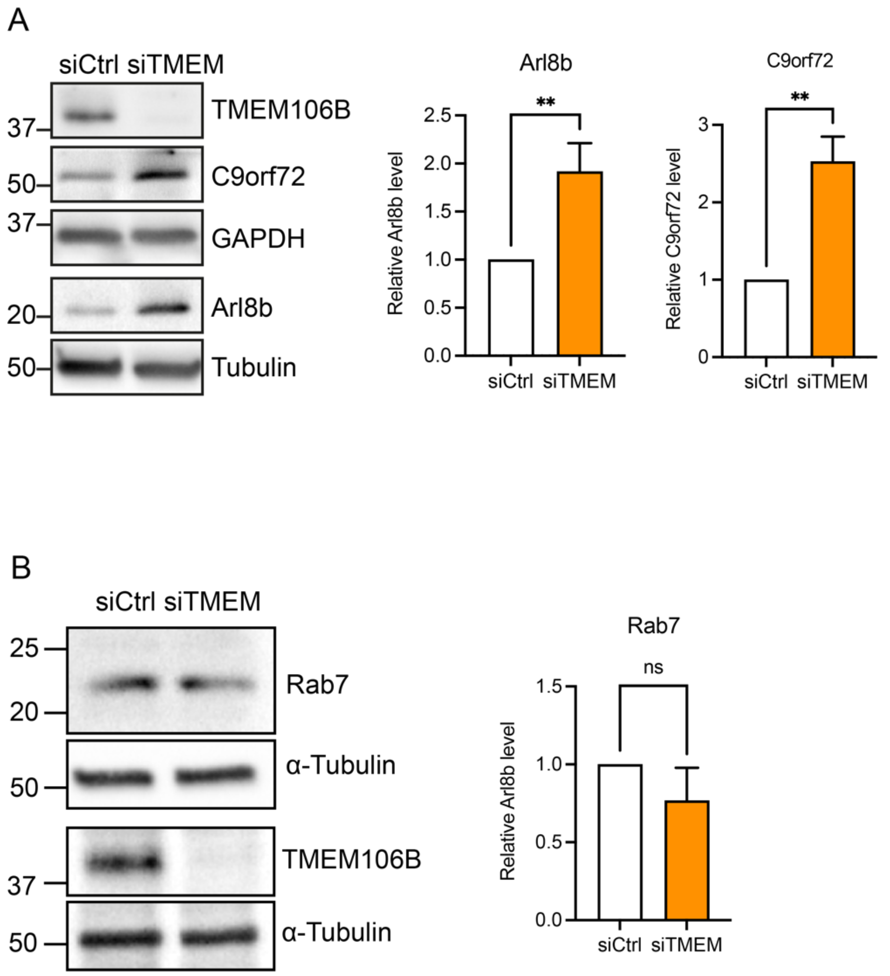

Supplementary Figure 8: Effect of TMEM106B knockdown on Arl8b and Rab7A levels.

(A) HeLa cells treated with either siCtrl or siTMEM106B were harvested and the level of TMEM106B, C9orf72 and Arl8b determined on immunoblot. C9orf72 and Arl8b levels were normalised against α-Tubulin and presented relative to siCtrl (mean ± SEM; unpaired t-test: $^{**}P \leq 0.01$; N = 9 experiments).

(B) HeLa cells treated with either siCtrl or siTMEM106B were harvested and the level of TMEM106B and Rab7 determined on immunoblot. Rab7 levels were normalised against α-Tubulin and presented relative to siCtrl (mean ± SEM; unpaired t-test: ns, not significant; N = 6 experiments).

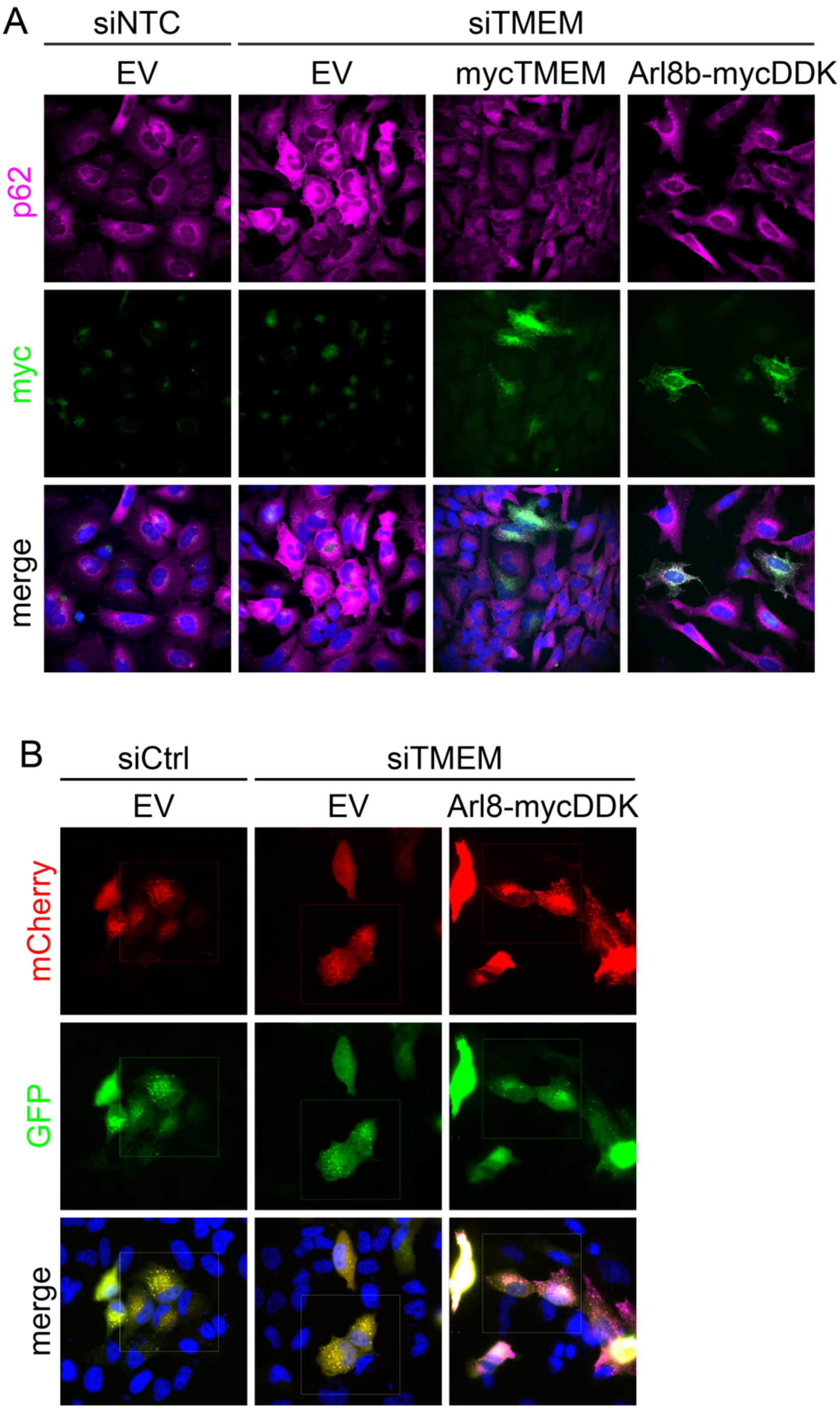

Supplementary Figure 9: Restoring Arl8b-mediated lysosomal trafficking rescues impaired autophagy in TMEM106B-depleted cells; full images of Figure 6. HeLa cells treated with either siCtrl or siTMEM were transfected with empty vector (EV), myc-tagged TMEM106B/T185, or mycDDK-tagged Arl8b (Arl8b-mycDDK). Cells were immunostained for endogenous SQSTM1/p62 (magenta) and the transfected myc-tag (green).

(B) HeLa cells were treated with non-targeting control (siCtrl) or TMEM106B targeted siRNA (siTMEM) for 3 days before transfection with mCherry-EGFP-LC3b and empty vector (EV) or Arl8b-mycDDK.

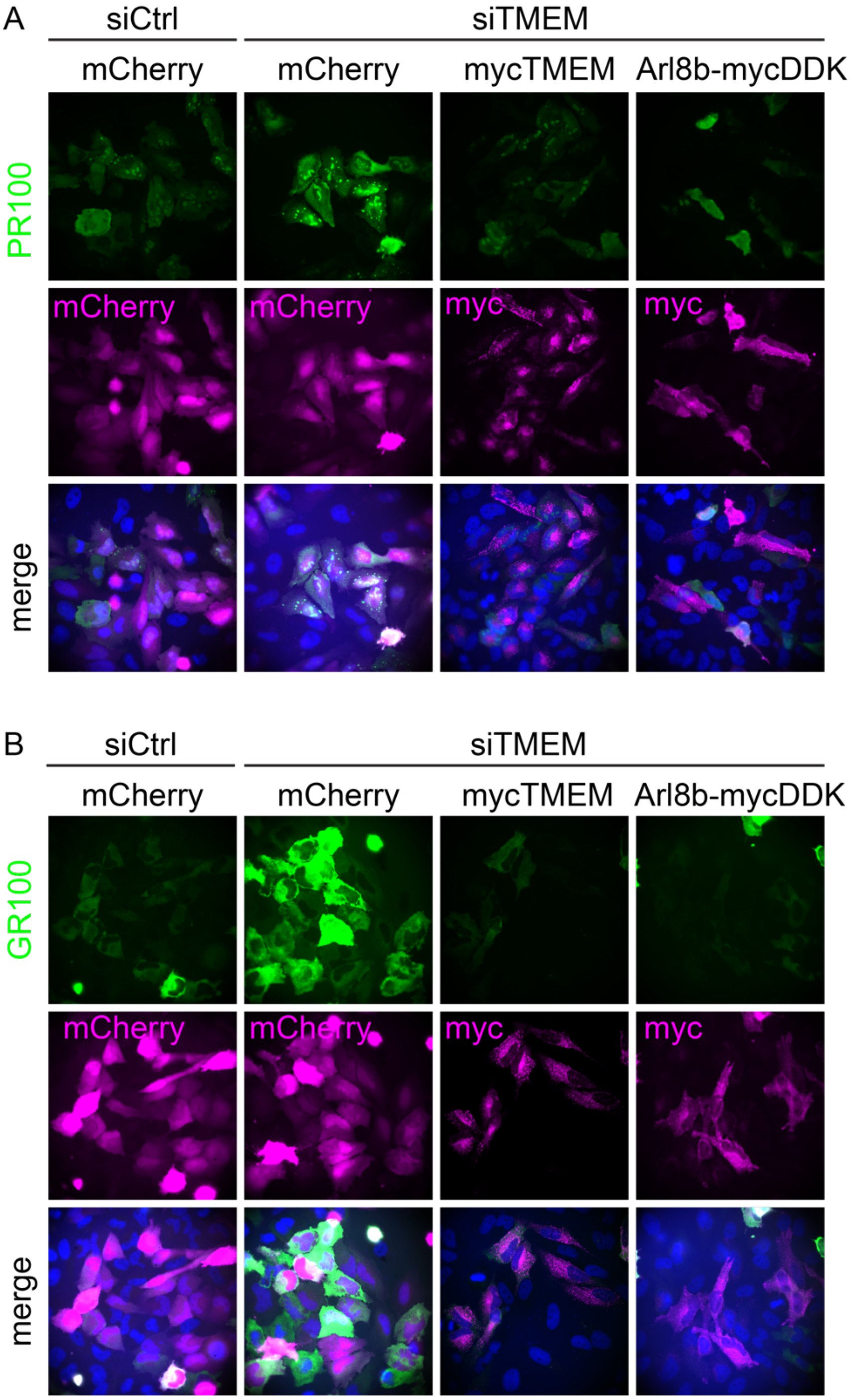

Supplementary Figure 10: Restoring Arl8b-mediated lysosomal trafficking rescues C9ALS/FTD DPR levels in TMEM106B-depleted cells; full images of Figure 7. HeLa cells treated with either siCtrl or siTMEM were transfected with mCherry, myc-tagged TMEM106B/T185, or mycDDK-tagged Arl8b (Arl8b-mycDDK) together with control mCherry (mCh), V5-tagged 100 repeat poly(PR) (PR100) (A) or V5-100 repeat poly(GR) (GR100) (B). Cells were immunostained for V5 (green) and myc (magenta); mCherry fluorescence (magenta) was visualised when present.

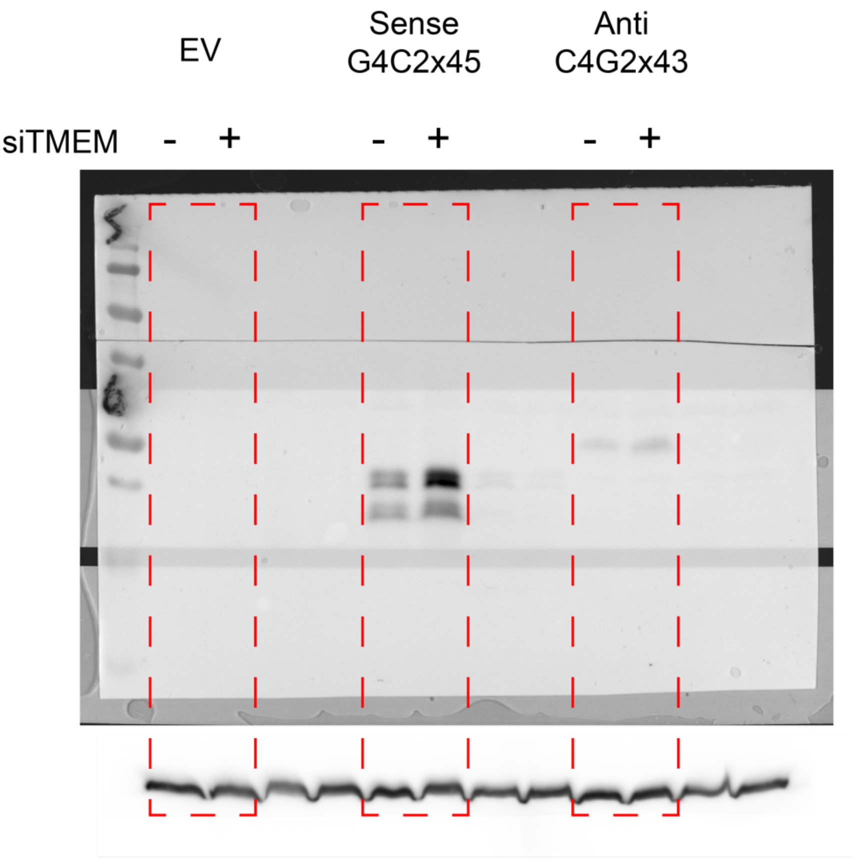

Raw

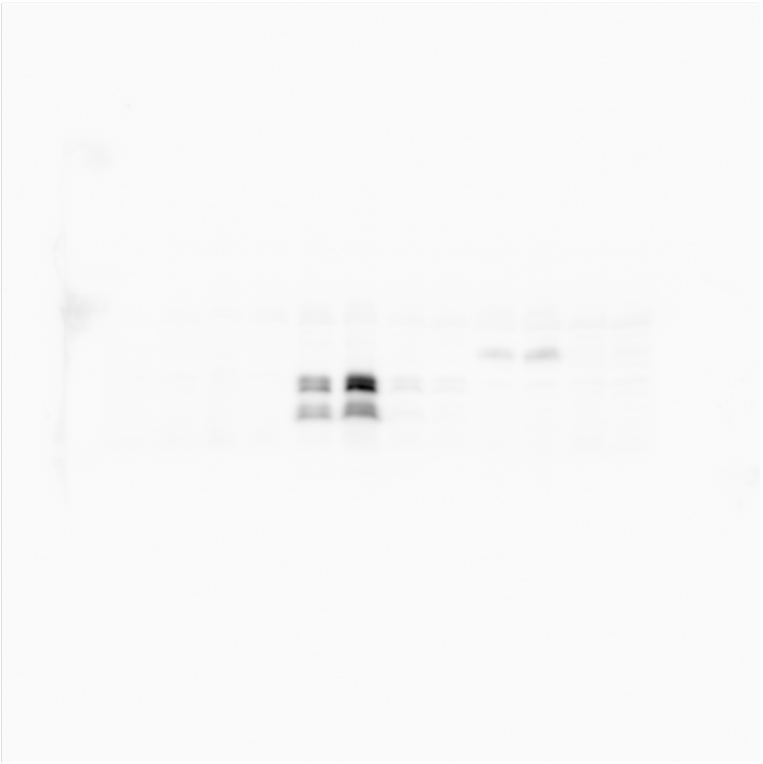

Supplementary Figure 11: Raw data of the immunoblots shown in Figure 1A. The top image shows the lanes cut out to make figure 1A.

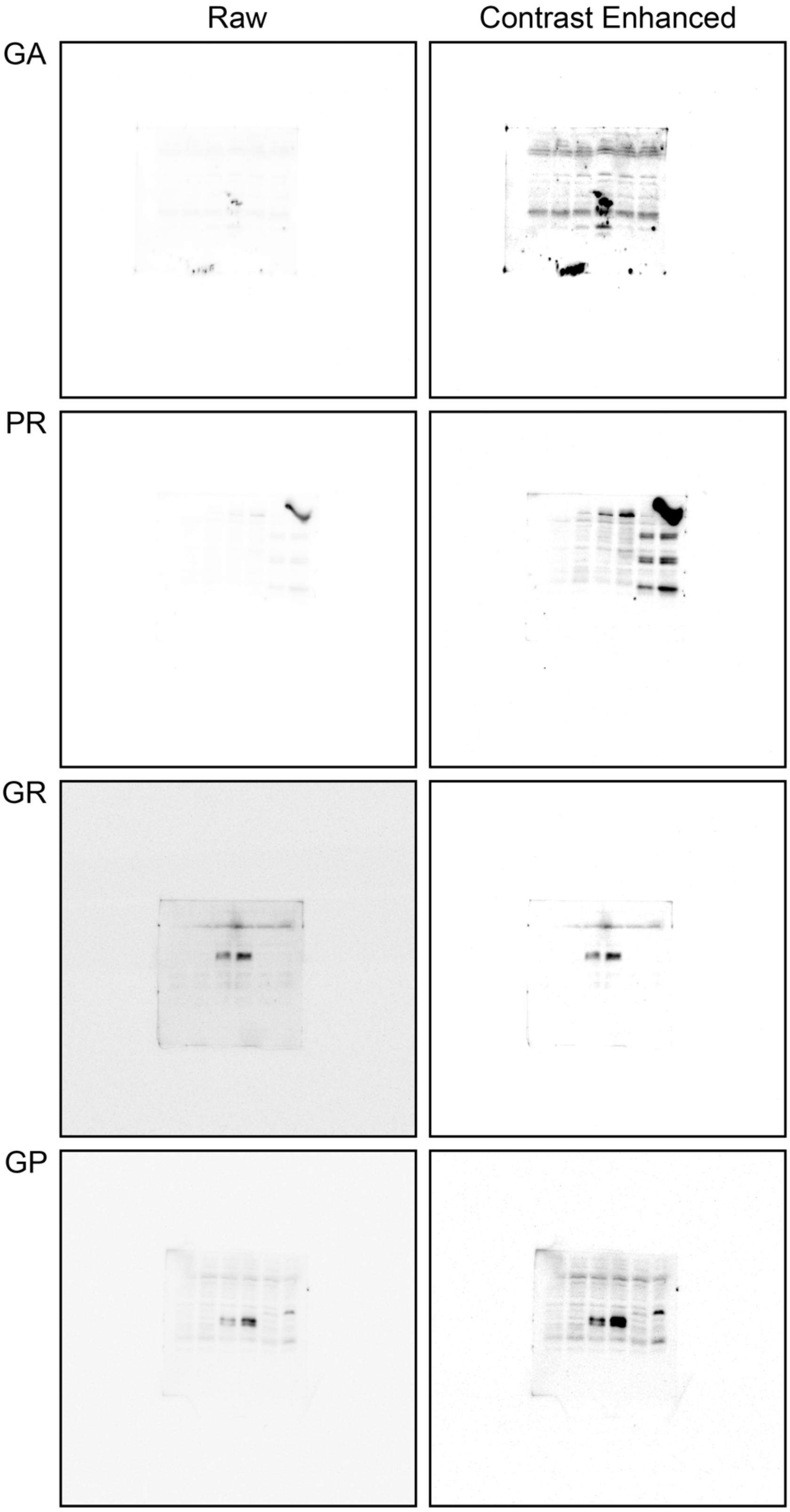

Supplementary Figure 12: Raw data of the immunoblots shown in Figure 1B.

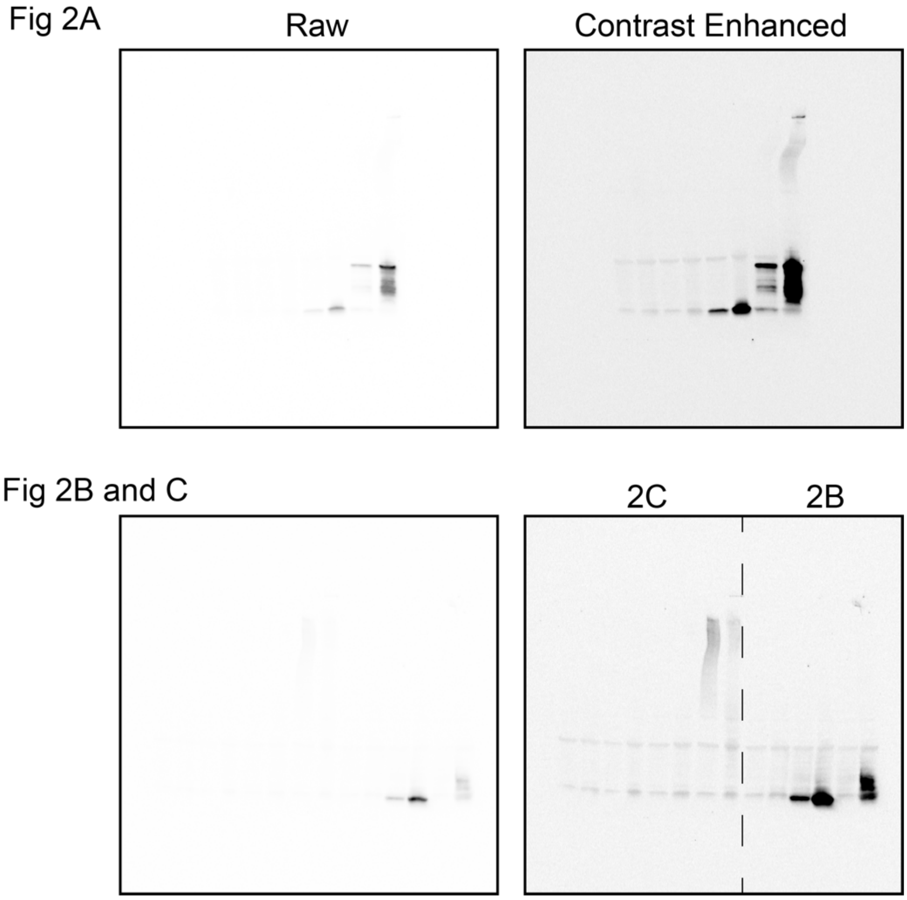

Supplementary Figure 13: Raw data of the immunoblots shown in Figure 2.
